# Short Conduction Delays Cause Inhibition Rather than Excitation to Favor Synchrony in Hybrid Neuronal Networks of the Entorhinal Cortex

**DOI:** 10.1371/journal.pcbi.1002306

**Published:** 2012-01-05

**Authors:** Shuoguo Wang, Lakshmi Chandrasekaran, Fernando R. Fernandez, John A. White, Carmen C. Canavier

**Affiliations:** 1 Neuroscience Center, Louisiana State University Health Sciences Center, New Orleans, Louisiana, United States of America; 2 Department of Bioengineering and Brain Institute, University of Utah, Salt Lake City, Utah, United States of America; 3 Department of Cell Biology and Anatomy, Louisiana State University Health Sciences Center, New Orleans, Louisiana, United States of America; École Normale Supérieure, College de France, CNRS, France

## Abstract

How stable synchrony in neuronal networks is sustained in the presence of conduction delays is an open question. The Dynamic Clamp was used to measure phase resetting curves (PRCs) for entorhinal cortical cells, and then to construct networks of two such neurons. PRCs were in general Type I (all advances or all delays) or weakly type II with a small region at early phases with the opposite type of resetting. We used previously developed theoretical methods based on PRCs under the assumption of pulsatile coupling to predict the delays that synchronize these hybrid circuits. For excitatory coupling, synchrony was predicted and observed only with no delay and for delays greater than half a network period that cause each neuron to receive an input late in its firing cycle and almost immediately fire an action potential. Synchronization for these long delays was surprisingly tight and robust to the noise and heterogeneity inherent in a biological system. In contrast to excitatory coupling, inhibitory coupling led to antiphase for no delay, very short delays and delays close to a network period, but to near-synchrony for a wide range of relatively short delays. PRC-based methods show that conduction delays can stabilize synchrony in several ways, including neutralizing a discontinuity introduced by strong inhibition, favoring synchrony in the case of noisy bistability, and avoiding an initial destabilizing region of a weakly type II PRC. PRCs can identify optimal conduction delays favoring synchronization at a given frequency, and also predict robustness to noise and heterogeneity.

## Author Summary

Individual oscillators, such as pendulum-based clocks and fireflies, can spontaneously organize into a coherent, synchronized entity with a common frequency. Neurons can oscillate under some circumstances, and can synchronize their firing both within and across brain regions. Synchronized assemblies of neurons are thought to underlie cognitive functions such as recognition, recall, perception and attention. Pathological synchrony can lead to epilepsy, tremor and other dynamical diseases, and synchronization is altered in most mental disorders. Biological neurons synchronize despite conduction delays, heterogeneous circuit composition, and noise. In biological experiments, we built simple networks in which two living neurons could interact via a computer in real time. The computer precisely controlled the nature of the connectivity and the length of the communication delays. We characterized the synchronization tendencies of individual, isolated oscillators by measuring how much a single input delivered by the computer transiently shortened or lengthened the cycle period of the oscillation. We then used this information to correctly predict the strong dependence of the coordination pattern of the firing of the component neurons on the length of the communication delays. Upon this foundation, we can begin to build a theory of the basic principles of synchronization in more complex brain circuits.

## Introduction

Several lines of evidence indicate that synchronous activity in the hippocampal formation is important for learning and memory. Coherent activity arises when animals are in states of active locomotion and information acquisition [1], [2]. Disabling coherent theta activity leads to memory impairment [3], [4]. Synchronous oscillations at gamma frequency have been implicated in binding of sensory experiences [5] and attention [6]. Computational models incorporating nested theta-gamma oscillations are well-suited to associative and sequence-learning tasks [7], [8], underscoring the potential importance of synchronous activity. Although many studies have analyzed systems of coupled oscillators, few [9] have incorporated the physical constraints of axonal conduction delays; therefore, there is a gap in our understanding of how distal neural modules can synchronize [10] that will be addressed in the proposed work. We use circuits constructed from stellate cells and pyramidal cells from the entorhinal cortex (EC) in rats in order to search for general principles of synchronization in the presence of conduction delays that may include multiple intervening synapses. Stellate cells in particular have been implicated as potential theta pacemakers [11].

Previously, Netoff et al. [12] used the Dynamic Clamp [13], [14] to measure the spike time response curves (STRC) for isolated layer 2 stellate cells in entorhinal cortex. The spike time response curve plots the change in cycle period due to a synaptic input as a function of the point in the cycle at which the input is received; in this study, we normalize the change in cycle period by the intrinsic period and call this the phase resetting curve (PRC). Using a strictly phenomenological criterion, Type I STRCs and PRCs contain either advances or delays whereas Type II contain both [15]. The STRCs (and PRCs) observed in response to excitation consisted of advances at most, but not all phases. The resetting was nearly zero at phases of zero and one with a peak near the center. There was a small region of small delays at very early phases; the presence of this region makes them weakly type II rather than Type I [15]. For inhibition, the PRCs consisted of only delays, hence they were Type I, but instead of having a peak in the center, the delays were monotonically increasing with phase. Netoff et al. [12] used the dynamic clamp to construct hybrid circuits of two biological neurons coupled by artificial synapses, or of one biological and one model cell. Their study, like ours, did not make any inferences regarding the response of the neurons to very weak pulses, but instead used the measured STRC directly to predict network activity, under the assumption that the pulsatile nature of the coupling made it likely that the effect of an isolated synaptic input was not changed by the mutual coupling within the network. The method successfully predicted that with no delays incorporated in the circuit, mutually excitatory circuits of stellate cells synchronized, whereas mutually inhibitory cells fired in antiphase.

Recently, Woodman and Canavier [16] derived existence and stability criteria for 1∶1 phase locking in a network of two oscillators reciprocally pulse-coupled with conduction delays. Pulse coupled means that the interaction between the coupled oscillators takes the form of brief pulses that can be approximated by delta functions with infinitesimal duration. The locking point for each oscillator is defined as the phase within its own cycle at which it receives an input from its partner during a one to one periodic locking in the network. For synchronous modes in circuits of identical oscillators, the phase at which an input is received by each oscillator is the delay divided by the intrinsic period of the oscillator, so increasing the delay above zero shifts the locking point along the PRC from zero phase to larger values of the phase. The key characteristic of the PRC that determines whether a 1∶1 locking such as synchrony is stable is the slope of the PRC at the locking point. Thus, depending upon the shape of the PRC, some delays will produce stable synchrony whereas others will not. Here we extend the work of Netoff et al. [12] on two coupled oscillators to include conduction delays, and to investigate the robustness of the synchronous solution to the heterogeneity and noise inherent in biological networks.

## Results

### Overview

**Figure 1:**
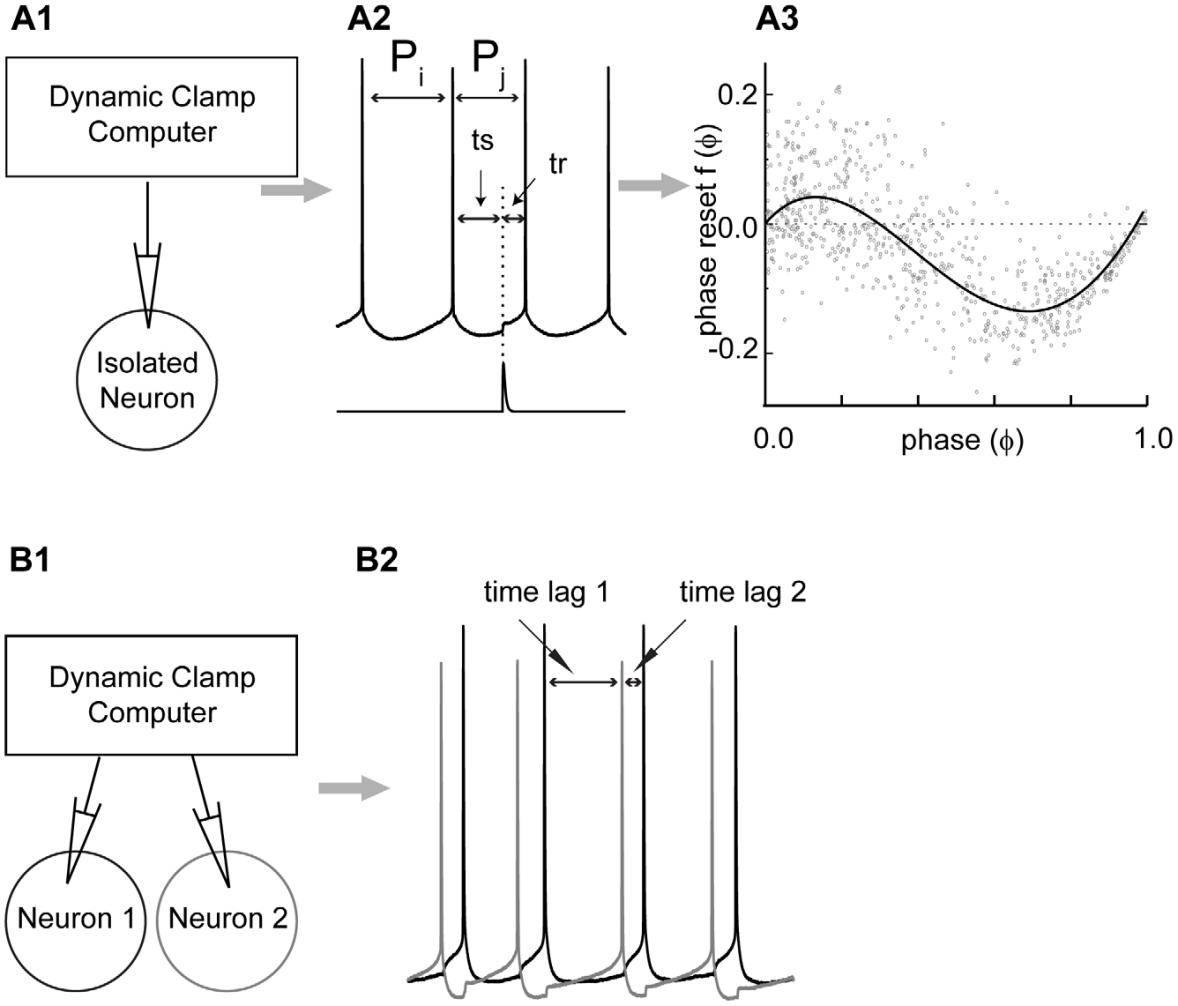
Measurement of the PRC and construction of hybrid circuits. A1. Dynamic clamp setup used to measure phase resetting curves in a pharmacologically isolated neuron. A2. Baseline current is applied to induce the neuron to fire repetitively (upper trace) then a simulated synaptic conductance (lower trace) is turned on after a stimulus interval ts and the next spike occurs after a recovery interval tr. The interval P_j_ containing the perturbation in general has a different length than the average unperturbed interval (P_i_). A3. The normalized change in cycle length (P_j_−P_i_)/P_i_ is called the phase resetting and is plotted versus the phase in the cycle at which the input was applied, calculated as ts/P_i_. The solid curve is a polynomial approximation of the mean phase resetting. B1. The dynamic clamp setup used to simulate synaptic conductances in two otherwise isolated biological neurons. Synapse activation was triggered by an action potential in the partner neuron, but a delay between the action potential and the delivery of the synaptic input to the partner neuron was programmed into the dynamic clamp. B2. Membrane potential recordings from hybrid circuits show alternating time lags in a one-to-one locking.

It is not practical to think that we can map out in detail the exact connectivity and characterize in detail every oscillatory element in brain circuits responsible for the synchrony that may underlie cognition. Instead, we seek to understand the general principles that underlie collective synchronous activity. Thus our general approach, illustrated schematically in Fig. 1, was to catalogue the representative characteristics of our circuit elements (EC neurons), then to use the range of characteristics of the individual components to predict and explain the range of collective activities observed when the individual components are connected in a network with conduction delays. To this end, we constructed very simple circuits in which we had complete control over the connectivity between the biological circuit components.

We used the Dynamic Clamp both to characterize the synchronization tendencies of individual neurons (Fig. 1A1) and to build simple networks (Fig. 1B1). The Dynamic Clamp is an electrophysiological method that allows one or more living cells to interface with a computer in real time. In the instances that spontaneous synaptic activity was observed in the biological neurons, the inputs were blocked pharmacologically (see Methods), and then virtual synapses were created as follows. The dynamic clamp sampled the membrane potential *V_mem_* in the soma of the biological neurons every 100 µs, then calculated and injected a synaptic current into each of the form *I_SYN_ = g(t)(V_mem_−E_syn_)* as described in the Methods. The same type of virtual synapse was used to characterize the phase response curve for each oscillator as the virtual synapses used in the hybrid networks. We measured PRCs using dynamic clamp experiments (Fig. 1A2) by applying either an excitatory or inhibitory synaptic current at various stimulus intervals (ts) after a spike to determine the recovery time (tr) until the next spike. The duration of the perturbed cycle (P_j_ = ts+tr) is in general different from the duration of the average free running unperturbed cycle P_i_. The normalized difference in cycle period is the phase resetting, which was calculated by the equation f_j_(φ) = (P_j_−P_i_)/P_i_, where P_j_ is the length of the cycle that contains the perturbation and plotted as a function of the phase at which the input was applied (Fig. 1A3). The phase (φ) is estimated by normalizing the stimulus interval *ts* by the average intrinsic period P_i_. The phase resetting was quite noisy, and curve fitting was used to determine the general shapes of the phase resetting that we can expect to encounter in these cells.

We then used the Dynamic Clamp to build simple two neuron networks (Fig. 1B1). The time-dependent synaptic conductance waveform *g(t)* was triggered in this case by a spike in the partner with an adjustable delay. Example voltage traces from a hybrid circuit experiment (Fig. 1B2) show a one-to-one locking in which there are two measurable time lags: the interval (time lag 1) between a spike in neuron 1 and the next spike in neuron 2, and the interval (time lag 2) between a spike in neuron 2 and the next spike in neuron 1. The average values of these time lags were measured for all pairs at different values of conduction delay between the neurons. Delays and time lags were normalized by the uncoupled period of the two neurons, which was set as nearly as possible to a single constant value using DC current (see Methods). The main goal of this study was to use the PRCs that were typically observed experimentally in order to account for the degree of synchronization actually observed in hybrid circuits, without knowing the exact PRC for each neuron in every circuit.

### Types of PRCs

**Figure 2:**
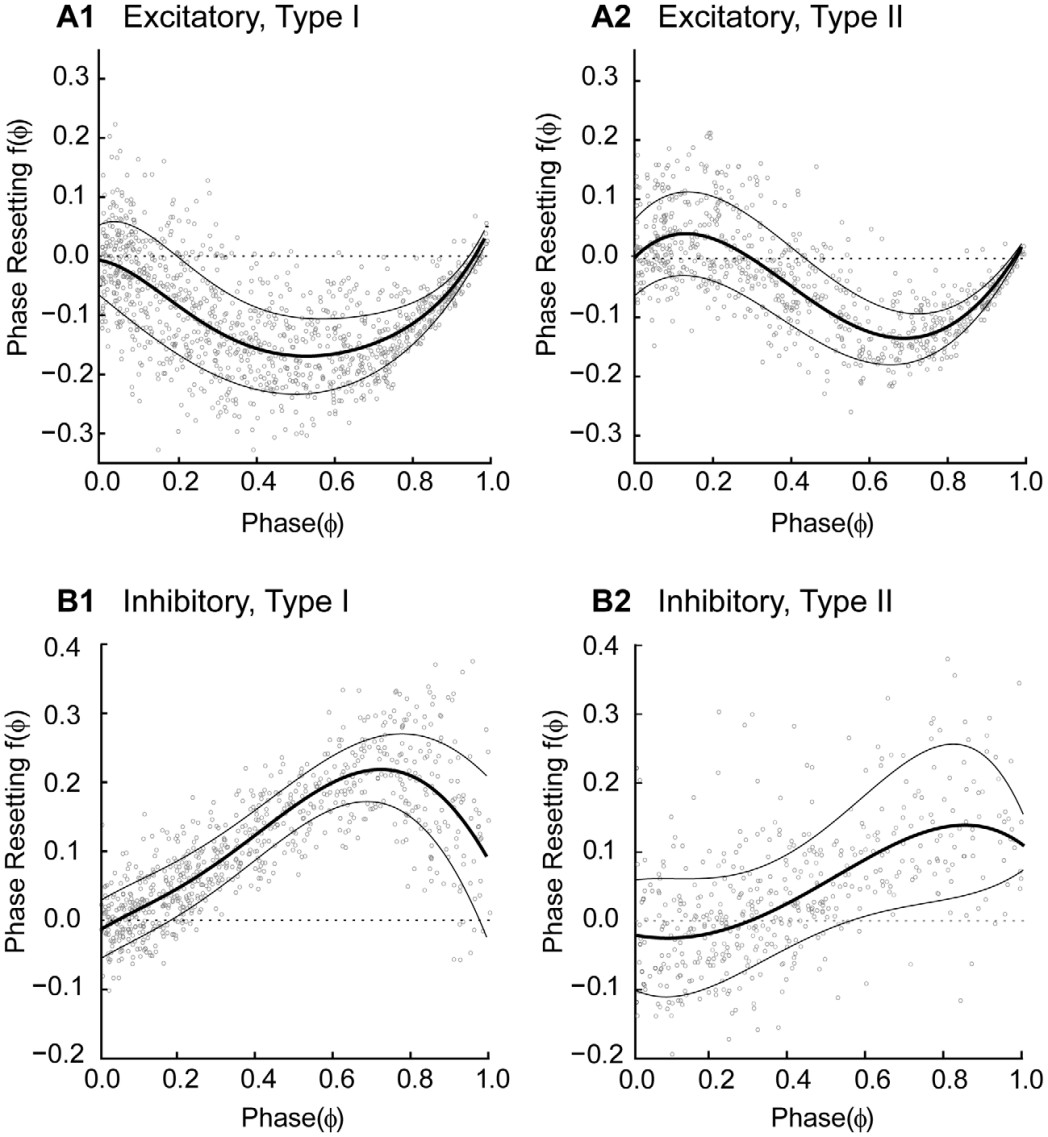
Typical PRCs measured with the Dynamic Clamp. In all cases, the best polynomial fit to the data is an estimate of the mean PRC (thick curve), and the envelopes (thin curves) are plotted one standard deviation above and below the mean to indicate how the variance in the data depends upon phase. A. PRCs in response to a virtual excitatory synapse. The variability of excitatory PRC decreases at late phases. A1. For Type I there is a single extremum indicating only advances. A2. Type II PRCs have more than one extremum and both delays and advances. B. PRCs in response to a virtual inhibitory synapse. The variability of inhibitory PRC is less phase-dependent. B1. For Type I there is a single extremum indicating only delays. A2. Type II PRCs again have more than one extremum and both delays and advances.

We measured a total of 24 PRCs (17 using a virtual excitatory synapse and 7 using an inhibitory one). Fig. 2 shows that two general classes of PRCs were observed for both inhibitory and excitatory coupling. In the convention used in this paper, a positive value of phase resetting means that the cycle period was lengthened, causing a delay before the next spike is emitted. A negative value corresponds to an advance in the time that a spike is emitted. Type I PRCs consist of either all advances or all delays, whereas Type II PRCs have a mix of the two [15]. Here we use these categories in a purely phenomenological sense, and make no implications regarding the excitability type [17] or bifurcation structure [18]. We found that for our data, the order of the best polynomial fit operationally allowed us to categorize Type I and Type II PRCs; those with a single extremum (implying a second order polynomial) in the best fit were consistent with Type I, whereas those with a higher order polynomial best fit were more consistent with Type II. Specifically, the 7 excitatory Type I PRCs exhibited only advances and a single inhibitory Type I PRC exhibited only delays. Ten excitatory Type II PRCs exhibited small delays at very early phases and advances at all other phases, and 6 inhibitory Type II PRCs exhibited small advances at very early phases and delays at all other phases. Thus the Type II PRCs were only weakly Type II. The best fit curve is an estimate of the mean PRC, and the mean plus or minus a single standard deviation is shown (thin curves) to give an idea of the phase dependence of the variability observed in the phase resetting. Consistent with [12], for inhibitory PRCs the variance was not strongly phase dependent, but for excitatory PRCs the variability decreased at late phases. Excitatory Type I PRCs had a negative slope at early phases, but a positive slope late, whereas the opposite was true for inhibition. Inhibitory PRCs did not appear to return to zero at a phase approaching one, also consistent with those reported by Netoff et al. [12]. Excitatory Type II PRCs had an initial and final positive slope but negative in the middle, whereas inhibitory Type II PRCs had an initial region of negative slope followed by a large region of positive slope. A second region of negative slope was sometimes observed at very late phases. The slopes have important implications for stability of phase locking; in our convention, negative slopes are destabilizing and positive slopes are stabilizing.

### Representative Experimental Results for Excitatory Hybrid Circuit

**Figure 3:**
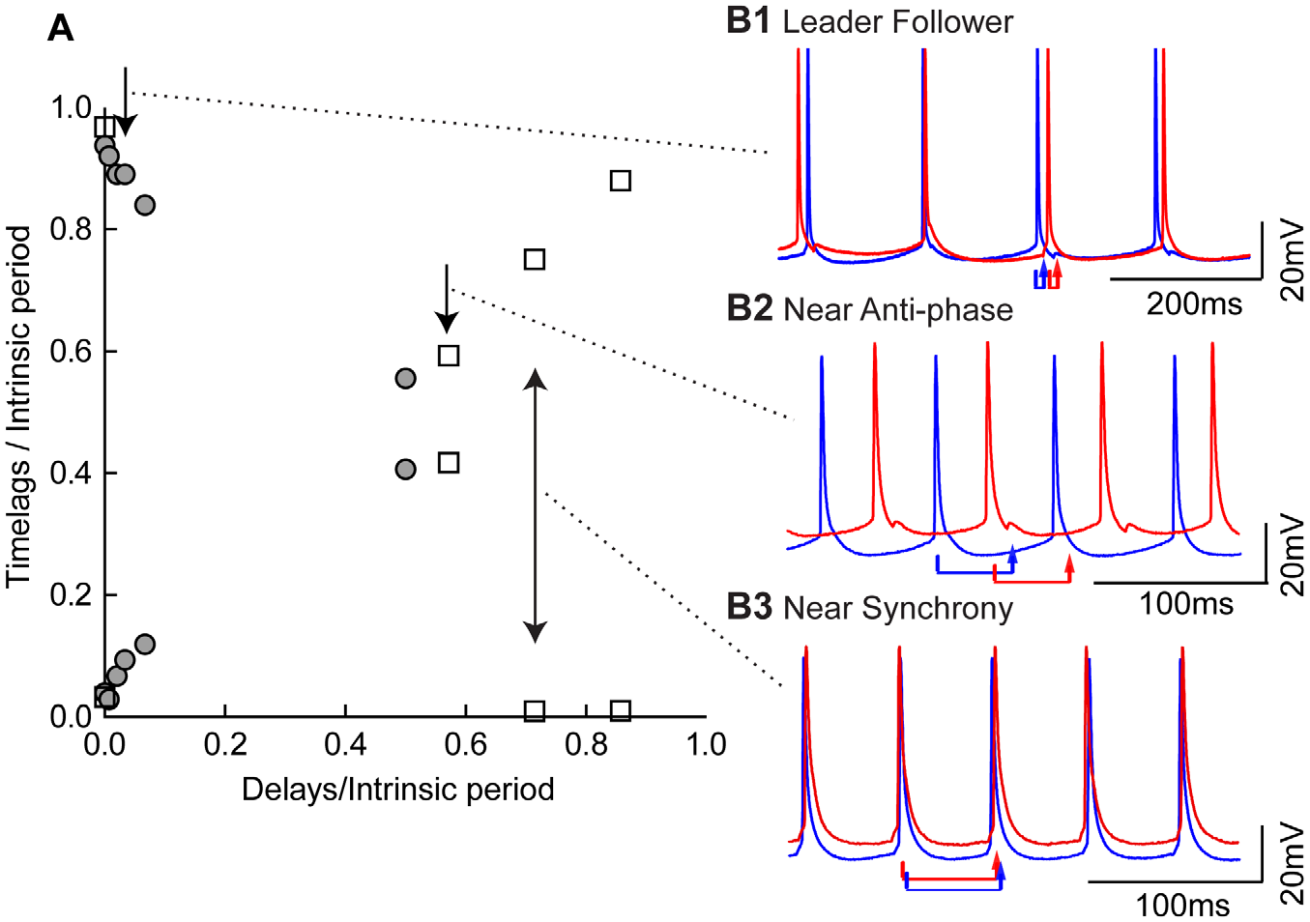
Typical firing patterns observed in excitatory hybrid circuits. A. Time lags observed in two hybrid circuits, one indicated by filled circles and the other by open squares, at different delay values. Due to constraints imposed by the duration that the experimental preparation remains viable, the full range of delays was not explored in any single circuit, but the type of patterns observed as the delay was increased was consistent across preparations. B. The red and blue arrows show the delay between action potential firing in one neuron and the arrival of an input to the other neuron. B1. Leader follower modes were observed at short delays (5 ms delay with intrinsic periods near 150 ms). B2. Near anti-phase modes were observed at intermediate delays (40 ms delay with intrinsic periods near 70 ms). B3. As delays were increased still further, a sharp transition to synchrony (one time lag near zero) was observed (50 ms delay with intrinsic periods near 70 ms).

Prior to turning the coupling on between two neurons, steady current was injected to cause the neurons to fire repetitively at similar frequencies (7∼10 Hz). Synchronization within a circuit was evaluated by constructing histograms of the time lags observed while the neurons were coupled via the dynamic clamp. Composite data in Fig. 3 from two excitatory hybrid circuits illustrate representative firing patterns observed in this type of circuit. Fig. 3A shows the peaks in the time lag histograms associated with the firing patterns illustrated in Fig. 3B. In a synchronous mode, one time lag is zero and the other is equal to the normalized network period. Synchrony was not in general observed in excitatory hybrid circuits with small delays. Instead, modes with one time lag that was roughly equal to the delay were observed at short delays. If the neurons are sufficiently homogeneous, then either neuron can lead, resulting in bistability between two firing patterns. We call this mode (Fig. 3B1) a leader/follower mode [19]–[21] because the firing of the leader evokes a spike in the follower (but not vice versa) after a delay equal to the time lag. In the first cycle, the red neuron leads, but leader switching is frequently observed, and the blue neuron leads in the last two cycles. Since the free-running periods of the two neurons were adjusted to be as nearly equal as possible, noise induced leader switches presumably due to bistability are not surprising. As the delays were increased to intermediate values, the leader/follower pattern transitioned to a near anti-phase mode (Fig. 3B2). At delays greater than half the period of the slower neuron, a sharp transition to synchrony was observed in which one value of the time lag was quite close to zero.

### Example of PRC-based Prediction Method

**Figure 4:**
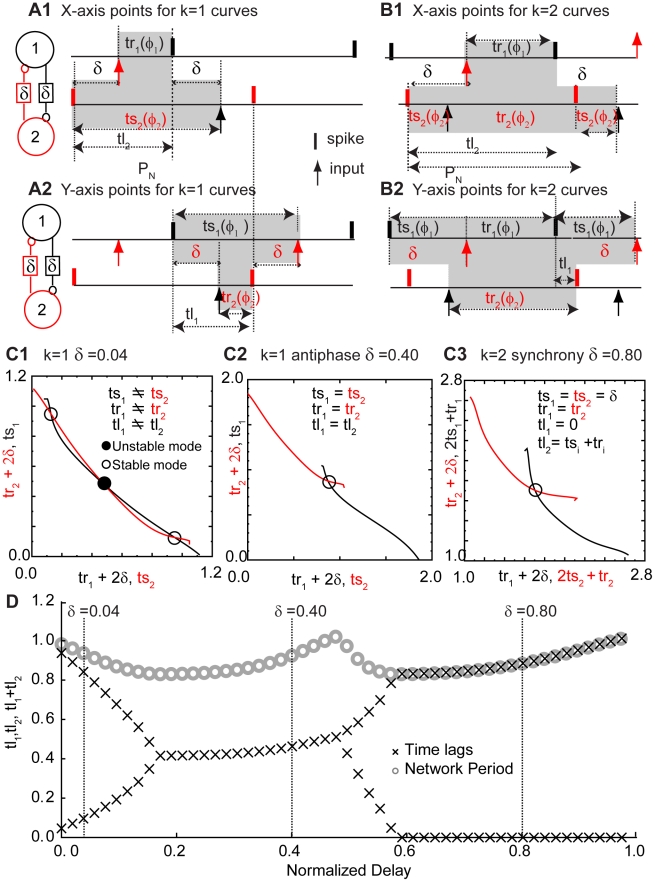
Graphical method for determining the periodic modes a two neuron circuit with conduction delays can exhibit. A. Periodicity constraints imposed by a pattern in which a spike in one neuron influences via a feedback loop the timing of the very next spike (k = 1) in the same neuron. B. Periodicity constraints imposed by a pattern in which a spike in one neuron influences via a feedback loop the timing of the second spike, but not the very next spike (k = 2) in the same neuron. C. Curves constructed, one for each neuron, for two identical neurons with a PRC as in Fig. 2A1, using the dependence of the stimulus and recovery intervals on the phase. The abscissa and ordinate points are reversed for one neuron as compared to the other so that intersections of the curves satisfy the appropriate periodicity constraints given in A or B. C1. At a normalized delay of 0.04, the open circles indicate unstable modes with two unequal time lags; either neuron can lead so there are two bistable modes. The dark circle indicates that the antiphase mode with two equal time lags is unstable. For stable points, the black curve is steeper than the red at the point of intersection. C2. For a normalized time lag of 0.40, the antiphase mode becomes stable as indicated by the open circle. C3. For normalized delays of 0.80, synchrony with one zero time lag becomes stable. D. The graphical method was applied at each value of the normalized delay in increments of 0.02. The time lags were calculated using the algebraic relationship of these quantities with the stimulus and recovery intervals shown in A or B as appropriate. Only time lags associated with stable modes (X symbols) were plotted. In addition, the network period, or sum of the time lags, was plotted as the gray circles.

Since there are two types of PRCs, a two-neuron circuit may be composed of two type I cells, two type II cells, or one of each. In order to determine if the observed activity could be explained using the PRCs, we used previously published theoretical methods [16] to predict the time lags corresponding to stable one-to-one lockings for each combination of phase resetting curves, using the representative examples from Fig. 2A and initially assuming that both neurons in the circuit had the same intrinsic period. We need to make the following assumptions in order to use PRCs to analyze network behavior of coupled neurons. 1) Each neuron is a pacemaker, i.e. a limit cycle oscillator, and remains so in the neural circuit. 2) The effect of single perturbation decays before the next input is received. This implies that the perturbed neuron returns immediately back to the limit cycle, otherwise the phase would be undefined when the input is received. 3) The perturbations that the neuron receives in a closed loop configuration are similar to ones that are used to generate the open loop PRCs. Given these assumptions, we can calculate the periodic one-to-one locked firing patterns that are consistent with the phase resetting tendencies of both neurons at any given value of conduction delay as illustrated in Fig. 4. The stimulus and recovery intervals can easily be calculated under these assumptions for any value of the phase *φ*, thus these intervals can be considered a function of the phase at which an input is received. The stimulus interval is *P_i_φ*, and from the definition of the phase resetting we can obtain that the recovery interval is *P_i_−P_i_φ+P_i_ f(φ)*.

Although we can calculate stimulus and recovery intervals for any arbitrary phase, only certain pairs of phases (*φ_1_*, *φ_2_*), where the subscript indicates the neuron receiving the input at a given phase, can satisfy the periodicity constraints for a one to one locking. In the presence of delays, it takes *k* cycles, where *k* is an integer, for the firing of one neuron to affect the next firing time in the same neuron. Fig. 4A1 and A2 illustrate the periodicity constraints for *k* = 1 and Fig. 4B1 and B2 illustrates them for *k* = 2. Briefly, twice the delay value plus the response interval (2δ+tr_i_) in one neuron by definition must be equal to the stimulus interval in the other neuron plus *k−1* times the network period (*ts_i_*+(*k−1*)(*ts_i_+tr_i_*)); this is true for both neurons resulting in two separate criteria that must both be satisfied in a one to one locking. For each neuron (neuron 1 in black and neuron 2 in red) we can plot these quantities at each phase as in Fig. 4C in order to find the intersections. The axes are selected so that at the intersections the abscissa and ordinate values for the red and black curves are equal so both periodicity conditions are satisfied. Furthermore, we can use the slopes of the phase resetting curve at the locking points to determine whether the firing patterns are stable and therefore observable in the presence of noise. If the slope of the black curve in Fig. 4C is steeper than that of the red curve at the point of intersection, the point is stable; otherwise it is unstable (see Fig. S1). The slopes of the PRC curve at the locking points determine the slopes of the red and black curves at the intersection points corresponding to one to one lockings; generally a positive slope of the PRC in our convention is stabilizing and a negative one is destabilizing (for an exact treatment see [16]). An intuitive explanation can be given for a slight perturbation from synchrony in a two neuron circuit; the neuron that fires too early receives an input at phase greater than the locking point, so for a positive slope at the locking point it is delayed more (or advanced less) than it would be at the locking point and therefore fires less early in the next cycle causing convergence to the locking point. The final step in the prediction method is to use the algebraic relationships depicted in Fig. 4A and 4B to determine the values of the time lags (see Fig. 4D) given the stimulus and recovery intervals and the delay values.

Fig. 4C and 4D specifically illustrate the PRC prediction method for two neurons with unit period and type I PRCs illustrated in Fig. 2 A1. At a delay that is 0.04 times the period (Fig. 4C1), there are three possible periodic one to one lockings, all with a *k* value of 1, as in the firing pattern shown in Fig. 4A; for that delay value, no other *k* values produce an intersection. The filled circle in the center is unstable and is ignored. The two open circles indicate modes in which the observable time lags are unequal. However, since the two neurons are identical, there are two firing patterns corresponding to these two time lags because either neuron can lead. This can account for the bistable leader follower mode observed experimentally in Fig. 3A. At a longer delay that is 0.40 times the period (Fig. 4C2), the antiphase mode with two identical time lags, again at a *k* value of 1, becomes stable. At even longer delays of 0.80 times the period (Fig. 4C3), the only intersection appears in the plot for a *k* value of 2, as in the firing pattern shown in Fig. 4B. This intersection produces a stable synchronous mode with one time lag equal to zero and the other equal to the network period *P_N_*, which is equal to the sum of the time lags (*tl_1_* and *tl_2_*) as well as the sum of the stimulus and recovery intervals in either neuron. The prediction results at each delay value are summarized in Fig. 4D. The X symbols show the values of the time lags calculated from the intersection points, and the gray circles show the network period, which is the sum of the times lags. For the antiphase mode at a delay of 0.40, for example, the two time lags overlay each other at exactly half the network period. Since this study was not limited to weak coupling, the network period can differ quite noticeably from the intrinsic period (assumed to be equal to one in this example) because of the resetting experienced by each neuron in the network. Consequently, the two points at each value of delay are not constrained to have a sum equal to one.

### Excitatory Hybrid Circuits: Observations Are Consistent with PRC-based Predictions

**Figure 5:**
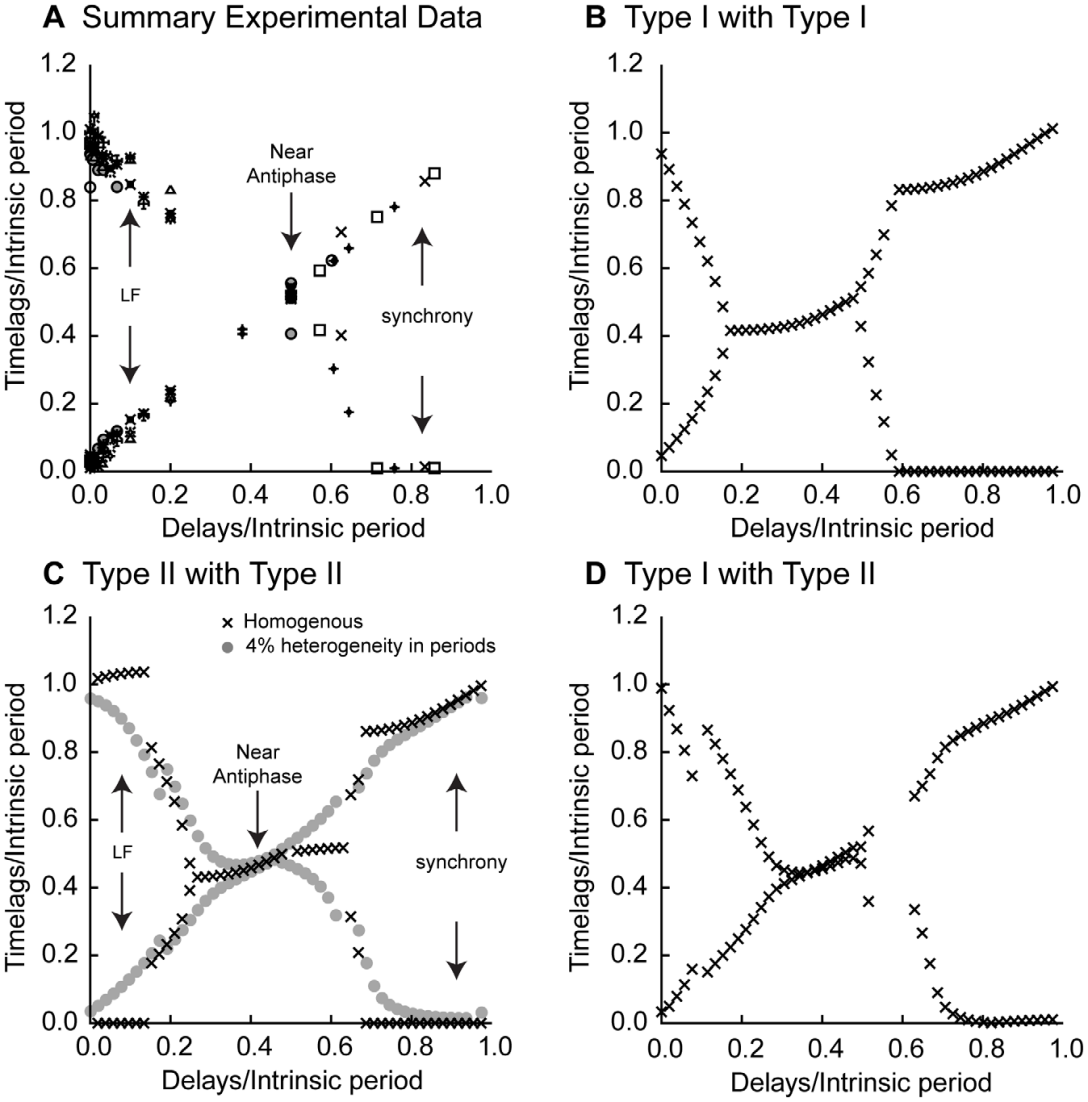
Experimental results are consistent with PRC-based predictions for excitatory hybrid circuits. A. Summary data from hybrid circuits (n = 11). The time lags and delays were normalized by the period of the slower neuron in the pair. Each symbol indicates a different hybrid circuit. As delays are increased, transitions from leader follower through antiphase to synchrony are observed. B. Predicted hybrid circuit activity for two identical cells with Type I PRCs as the delay is varied (same as Fig. 4D without the network period. C. Predicted hybrid circuit activity for two identical cells with Type II PRCs as the delay is varied. The filled circles show how the solution structure is disrupted by a 4% difference in intrinsic period between the component neurons. D. Predicted hybrid circuit activity for two cells with the same period but in this case one has a Type I PRC and the other has a Type II PRC. Note: The absence of symbols at a particular delay in Fig. 5A indicates that those delays were not sampled experimentally. On the other hand, the absence of symbols at the regularly sampled intervals in Fig. 5D indicates that no stable modes were predicted at those delays.

A total of twelve hybrid circuits were constructed from eight pairs of biological neurons coupled by excitation, of which four pairs were coupled at two different conductance values. Clear peaks in the histograms indicating one-to-one phase locking with preferred time lags were evident in all but one experiment. We excluded the data from that experiment, which happened to be from one of the pairs in which experiments were conducted at two conductance values. The circuit that did not lock had the weakest conductance value used in any experiment, and was apparently too weak to induce phase locking at any value of delay recorded. Data from the eleven phase-locked circuits is summarized in Fig. 5A, using a different symbol for each circuit. The summary data shows the same dependence of the observed firing pattern on the conduction delay that was clearly illustrated in Fig. 3. We then compared the results of the PRC prediction method for all possible combinations of PRC type. The predicted values of the normalized time lags (X symbols) for each of the three cases are plotted in Fig. 5B, C and D. For the circuits that contain at least one Type I neuron (Fig. 5B and D), the predicted activity follows the same trends as the experimental data in Figs. 3A and 5A. In particular, Fig. 5B and D show prominent leader-follower behavior for normalized delays less than 0.2–0.3. For circuits of two identical Type II neurons, synchrony rather than leader follower mode was predicted for short delays, but if the periods were allowed to vary by a few percent (4% in the circles in Fig. 5C), then early synchrony was disrupted and the general trends observed in the experimental data, including an approximate leader/follower mode at short delays, were restored. Therefore the experimental results for excitatory hybrid circuits are quite consistent from what is expected using PRC theory.

**Figure 6:**
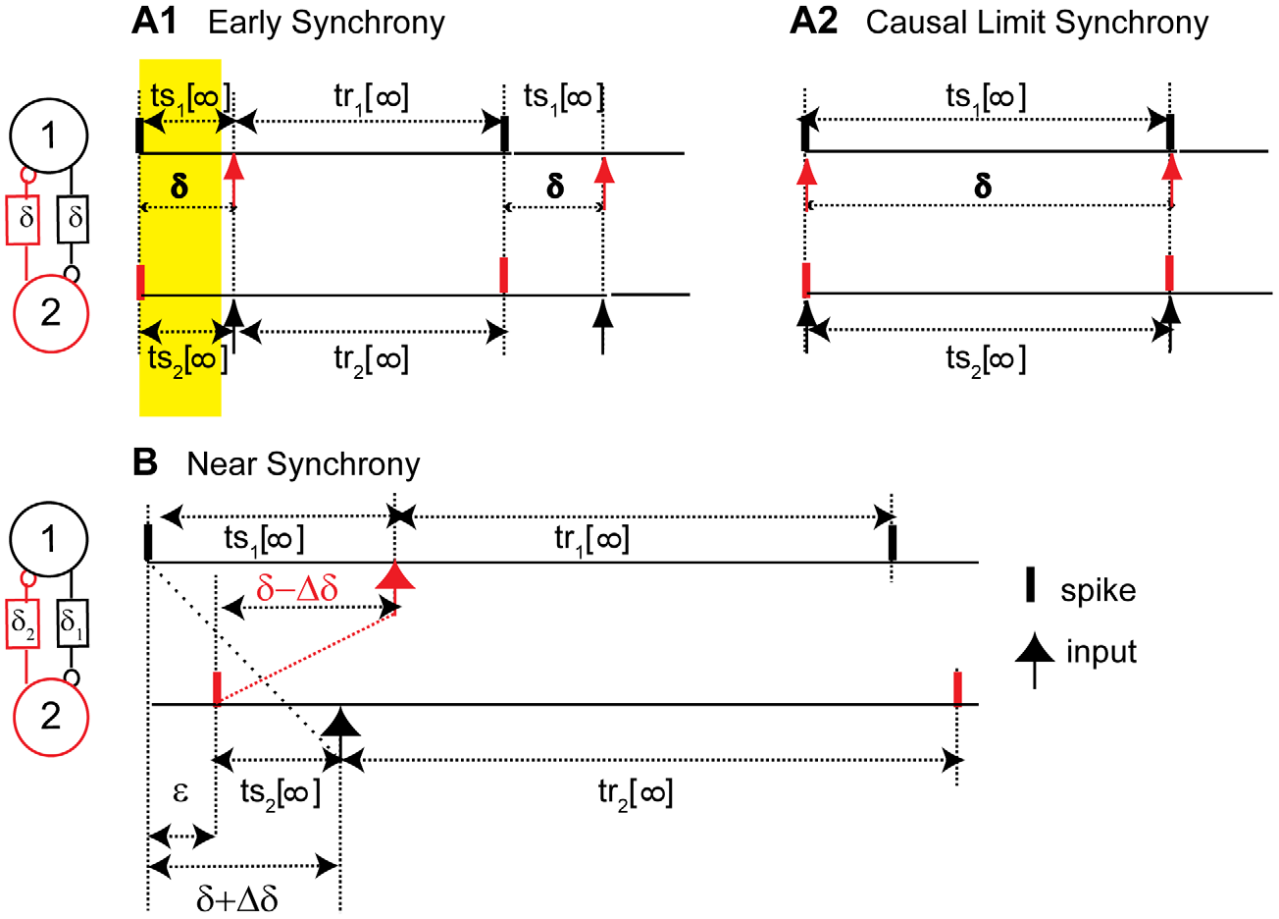
Relationship of the delay to the locking point in the synchronous mode. A. For a periodic one to one locking, the steady state values of the stimulus (ts_i_) and recovery (tr_i_) intervals is indicated by the index [∞]. A1. For a circuit of two identical neurons with identical conduction delays δ, if the two neurons fire at the same time, then each neuron receives an input at a phase of δ/P_i_, where P_i_ is the intrinsic period of neuron i. A2. As the recovery interval shrinks to its theoretical limit of zero, the phase at which an input is received is still δ/P_i_ the network period is now equal to the delay, which was not the case for shorter delays. B. Slightly different intrinsic periods, conduction delays or both perturb exact synchrony such that there is a small time lag ε between the firing of the neurons. If the perturbed locking points remain in a nearly linear neighborhood of the locking point for the homogeneous circuit, then an exact expression can be derived for ε (see text and derivation in Text S1).

More importantly, the use of the PRC methods allows us to gain insight into why the observed firing patterns are favored. Specifically, synchrony is only observed for zero delay and delays that are longer than half the intrinsic periods. First we will discuss why synchrony is not observed at short delays, and then we explain why it is observed for longer ones. With no delay, the locking point for synchrony is at a phase of zero because each neuron affects the other immediately upon spike initiation at a phase of zero. As the delay is increased, the locking point is moved to the right along the PRC [16] to a phase equal to the delay divided by the intrinsic period (see Fig. 6A). The slope of the Type I PRC shown in Fig. 2A1 is negative for over half the cycle and therefore destabilizes synchrony for delays less than half an intrinsic period. Thus we would not expect synchrony for short delays in any circuit that contains a Type I PRC. On the other hand, the Type II PRC shown in Fig. 2A2 has an early region of stable positive slope for phases less than about 0.15, so we might expect synchrony in the cases in which the hybrid circuit happens to contain two neurons with Type II PRCs. This synchrony, however, results from the symmetry of two identical, identically coupled oscillators [22], and was easily disrupted in Fig. 5C by the introduction of heterogeneity in the period. It is striking, however, that for all panels in Fig. 5, long delays (greater than 0.6 to 0.8 of the intrinsic period depending upon the specific example) produced robust synchronization that was not disrupted by heterogeneity either in the hybrid circuits (Fig. 5A) or in the coupled PRCs (Fig. 5C). Not coincidentally, this robust synchrony occurs when the locking point nears the causal limit region of the PRCs towards the end of a cycle when an excitation almost immediately evokes a spike. Thus the magnitude of the phase advance is equal to the fraction of the cycle remaining at the time the input is given (1−φ). Under our sign convention, this produces a linear region (φ−1) in the PRC with a positive slope of one that is strongly stabilizing.

### Effects of Heterogeneity on Synchrony: Theoretical Results

In order to explain why early but not late synchrony was disrupted by heterogeneity, we quantified the degree to which small deviations from synchrony caused by heterogeneity can be quantified in terms of the PRC. Assuming that the PRC for two neurons is identical, but that they have a small difference in intrinsic period, we can derive this expression for the nonzero time lag ε when the synchronous solution is disrupted by the unequal intrinsic periods P_1_ and P_2_ and a difference Δδ in the delays δ, where δ represents the average of the two delays:
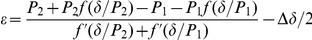
where *f* is the phase resetting and the prime indicates the slope. This expression is valid for small ε+Δδ/2 so that the PRC can be linearized (see Fig. 6B and the derivation in Text S1). For identical neurons, the numerator in the fractional term is zero, which allows exact synchrony for equal delays (Δδ = 0). In Fig. 5C, equal delays but non-identical intrinsic periods cause a nonzero phase lag in the near synchronous solution (circles versus crosses). Increasing the slope of the phase resetting curve by increasing the conductance is not an effective strategy to minimize the time lags because very large positive slopes are also destabilizing. For the synchronous solution, the absolute value of one minus the sum of the slopes at the two locking points (one for each neuron) needs to be less than one for stability [16], [23]. At late phases for strong coupling, the PRCs in the vicinity of the locking point are essentially the same and linear at the causal limit, so the expression given above for the nonzero time lag is quite valid. At the causal limit, f(δ/P_i_) = δ/P_i_−1 and the numerator goes to zero even with different intrinsic periods or PRCs as long as the locking point for both is on the causal limit. Because the slope of each PRC is one in this region, the time lag ε reduces to (Δδ)/2, which is zero for identical delays. Exactly on the causal limit, synchrony becomes neutrally stable in theory; however, the causal limit cannot be physically achieved because some time must elapse between an action potential in the leader and the one it evokes in the follower.

### A Noisy PRC-based Map Explains the Robustness of Near-Causal-Limit Synchrony to Noise

**Figure 7:**
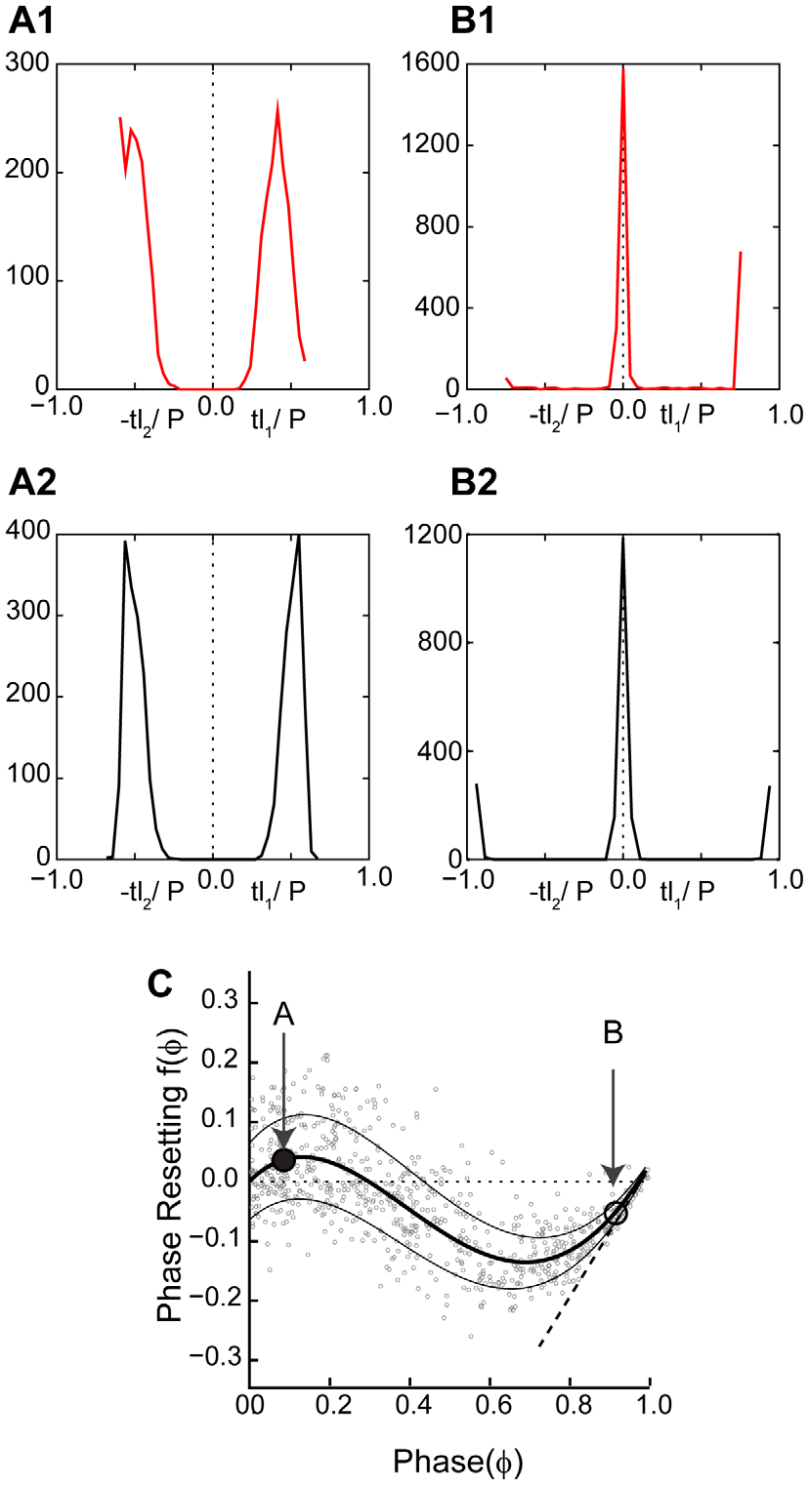
Noisy map based on the PRC accounts for the tight synchrony near the causal limit. Histograms from the hybrid circuit shown in Fig. 3B2 with a delay of 40 ms (A1) and in Fig. 3B3 with a delay of 50 ms (B1), relative to an intrinsic period of about 70 ms. Histogram peaks for the synchronous mode at the longer delay are much narrower than for the antiphase mode. Histograms were also generated by a noisy map based on a hypothesized circuit composition of two cells with Type II PRCs as in (C). For a normalized delay of 0.55 a histogram with two wide peaks (A2) corresponding to an antiphase mode results. As the normalized delay is increased to 0.9, an abrupt transition to synchrony with a much narrower peak (B2) is observed. C. The locking point for antiphase (filled circle marked A) falls in a much noisier region of the PRC than the locking point for synchrony (open circle marked B). The dashed line indicates the causal limit.

We previously mentioned that an abrupt transition to synchrony (Fig. 3A) was observed between intermediate (Fig. 3B2) and long delays (Fig. 3B3). This abrupt transition as the delay was increased was sometimes accompanied by an abrupt increase in the tightness of the phase locking. Fig. 7A1 shows the histogram of the times lags for the antiphase mode illustrated in Fig. 3B2 in a hybrid circuit with a normalized delay of about 0.57. Rather than plotting both times lags on the same axis, as in Figs. 3A, 4D and 5, here we have plotted time lag 1 (see Fig. 1B2) as positive and time lag 2 as negative so that we get two distinct peaks for antiphase. Circular statistics (see Methods) showed that the circuit was locked at a network phase of 0.5 with an R^2^ = 0.7. Fig. 7B1 shows a histogram of the time lags for the synchrony illustrated in Fig. 3B3 at a normalized delay of about 0.71. The histogram for synchrony has a peak at zero and peaks at ± the network period depending on which neuron is considered to fire first, but in this case one peak is smaller than the other indicating that the faster neuron fired first more often, breaking symmetry. The peaks for synchrony had a narrower width indicating tighter locking than in the antiphase example, as confirmed by circular statistics indicating a network phase of 0 for synchrony with an R^2^ = .87.

The transition from antiphase to synchrony is evident in each panel of Fig. 5 where a clear antiphase mode with equal or roughly equal time lags at intermediate delays is replaced by synchrony as the delays are lengthened by at most 10% or 15% of the period from the clear antiphase mode. We can explain the narrowing of the histogram peaks by assuming that in this example, both cells in the hybrid circuit had identical Type II PRCs as in Fig. 7C. In this hypothetical circuit, for an antiphase mode the locking point for the individual is not at 0.5 phase but rather at the phase that satisfies *φ = 0.5+f(φ)/2+δ/P_i_*, because of contributions from nonzero phase resetting and from the conduction delays. For a normalized delay of 0.55, the locking point corresponding to antiphase has shifted far enough to the right to “wrap around” a phase of one and land on the initial stable branch of the PRC with positive slope (filled circle labeled A in Fig. 7C). This branch is quite noisy. As the normalized delay is increased, antiphase loses stability as the locking point moves onto the middle unstable branch of the PRC. The locking point for the individual neurons in a synchronous mode is not at zero phase, but rather at the normalized delay value. As the normalized delay value increases to 0.9, the locking point for synchrony (open circle marked B in Fig. 7C) falls in the causal limit region of the phase resetting curve (dashed line) where an excitatory input reliably triggers a spike with short latency, reducing the noisy variability.

We suspected that the sudden decrease in the width of the histogram of network phases observed in the transition from antiphase to synchrony (Fig 7A1 compared to 7B1) could be accounted for by a switch in the locking point on the PRC from a region of high variability to a region of lower variability. In order to test this possibility, we constructed a noisy iterated map (see Methods) based on the PRC [12], [24] by initializing each neuron in a simulated hybrid circuit at an arbitrary phase, polling the neurons to see which one would fire next, updating the phase of the partner to the firing time, keeping track of input emission and delayed arrivals, and resetting the phases appropriately when an input arrived. The phase resetting was a random Gaussian variable with the mean and the variance determined at each phase by the experimental data. Previous such maps [12], [24] did not include the greater complexity encountered in the presence of delays. The noisy map produced the broad histogram peaks shown in Fig. 7A2 for the antiphase mode with a normalized delay of 0.55. Circular statistics gave an R^2^ = .86 at a network phase of 0.5. On the other hand, the noisy map produced the narrow histogram peaks shown in Fig. 7B2 for the synchronous mode with a normalized delay of 0.9. Circular statistics gave an R^2^ = .98 at a network phase of 0, confirming that the phase dependent variance of the PRC and specifically the decrease in the variance at very late phases, can provide a possible explanation for the tighter phase locking that is sometimes observed in the transition to synchrony as the delay is increased.

### Inhibitory Hybrid Circuits: Observations Are Consistent with PRC-based Predictions

**Figure 8:**
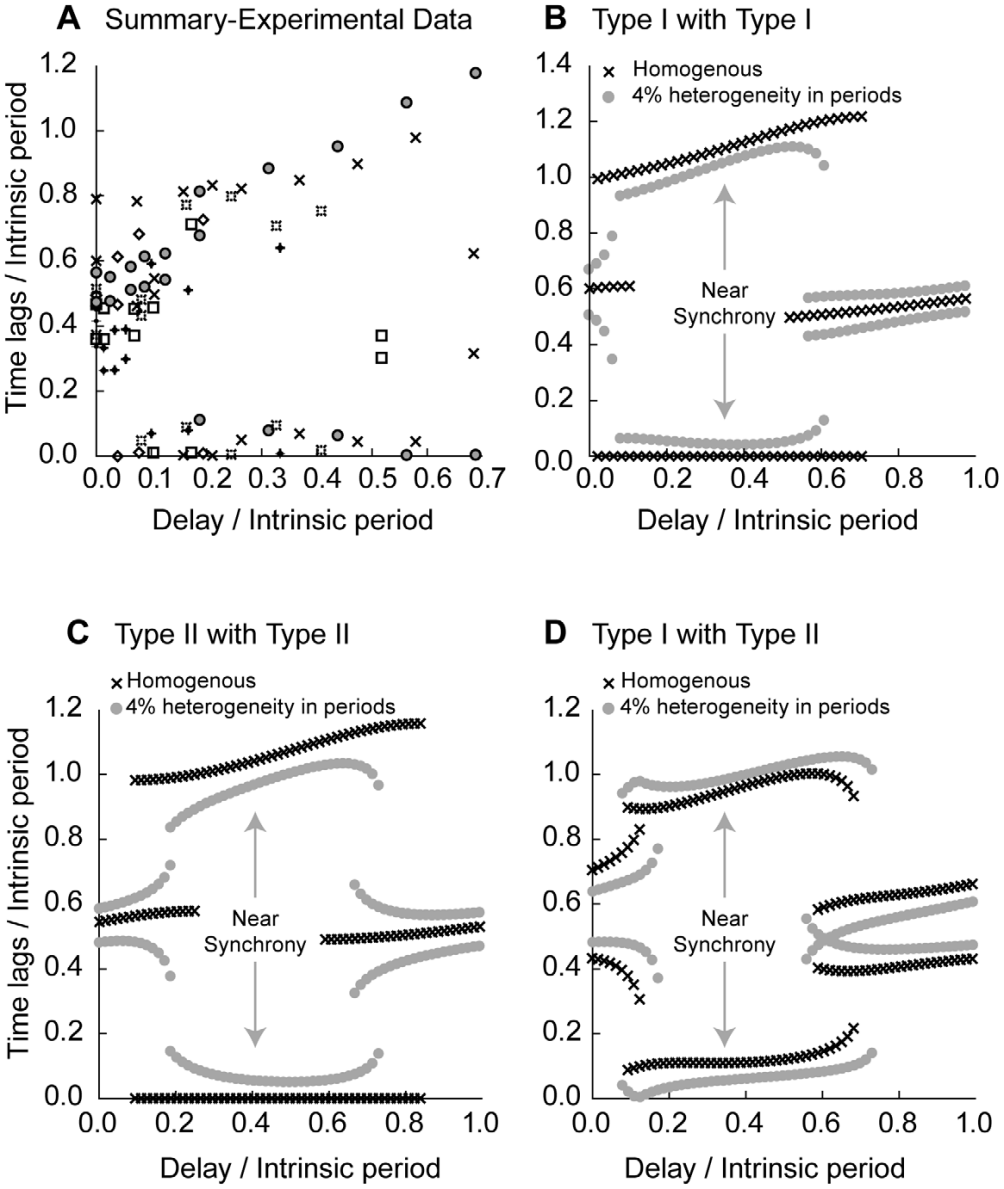
Experimental results are consistent with PRC-based predictions for inhibitory hybrid circuits. A. Summary data from six inhibitory hybrid circuits. The time lags and delays were normalized by the period of the slower neuron. Each symbol indicates a different hybrid circuit. B. Predicted hybrid circuit activity (X symbols) for two identical Type I PRCs (see Fig. 2 B1) as the delay is varied. The predicted time lags with 4% heterogeneity in period (filled circles) C) Predicted hybrid circuit activity (X symbols) for two identical Type II PRCs (see Fig. 2B2) as the delay is varied. The predicted time lags with 4% heterogeneity in period (filled circles) D) Predicted hybrid circuit activity for a Type I PRC with a Type II PRC with identical periods (X symbols) or with 4% heterogeneity in period (filled circles) as the delay is varied.

Fig. 8A shows the summary data for 6 hybrid circuits coupled by inhibition. The tendency was to exhibit antiphase at the shortest delays and near synchrony at slightly longer delays of up to 0.7 times the intrinsic period, the largest values explored in these circuits. At the longest delays examined, out of the three cell pairs tested at these delays, two pairs (X symbols and open squares) exhibited a transition from near synchrony to near anti-phase as the delay was increased, suggesting that if longer delays were applied the other cell pairs would have undergone the transition as well. Many of the time lags are quite close to zero, across a broad range of phases including relatively early phases, in contrast to the hybrid circuits coupled by excitation. Once again we used each possible combination of PRC type within a circuit to predict how the observed time lags should vary as the delay is increased, and the predicted pattern of the dependence of network activity on the delay shown in Fig. 8B–D conforms to the overall pattern obtained experimentally in Fig. 8A. For a circuit with two identical neurons with Type I PRCs (X symbols in Fig. 8B) synchronous modes are predicted for a large range of phases corresponding to normalized delays from 0 to 0.75, because the slope of the Type I PRC (Fig. 2B1) is positive in that range. Introducing heterogeneity in the form of a 4% difference in cycle period only slightly perturbs the synchronous solution (gray filled circles in Fig. 8B), except at very short normalized delays (<0.1). For some normalized delays (less than about 0.1 and between 0.5 and 0.7), antiphase is bistable with synchrony, so either mode could theoretically be observed depending upon the initial conditions. The same level of heterogeneity disrupts early but not late bistability. For a circuit with two identical neurons with Type II PRCs (X symbols in Fig. 8C), synchronous modes are predicted at phases between about 0.1 and 0.85 because the slope of the Type II PRC is positive in that region. There is also a region of bistability with antiphase from about 0.1 to 0.3 and from about 0.6 to 0.85. Heterogeneity in the form of a 4% difference in intrinsic periods did not severely disrupt synchrony, although synchrony in the Type I circuits in Fig. 8B was more robust than the Type II in Fig. 8C. This level of heterogeneity reduces but does not eliminate the bistable regions. The predicted time lags in Fig. 8B and Fig. 8C are in qualitative agreement with the summarized experimental results in Fig. 8A. The final possibility, a circuit with one cell with a Type I PRC and other with a Type II PRC, does not synchronize at any delay when the periods are matched (predicted time lags indicated by X symbols in Fig. 8D), so we conclude that this type of circuit was not represented in the hybrid circuits that we constructed. Nonetheless, there was a region in which the time lag was fairly flat over a range of delays (about 0.1 to 0.6). If heterogeneity in the periods is introduced by slowing down the Type II neuron, this has the effect of matching the network periods because the Type II neuron is in general delayed less (or advanced more) than the Type I neuron at the same phase. This in turn pushes the circuit toward synchrony (filled gray circles in Fig. 8D) and is an alternate, but less likely explanation, of why near synchronization was universally observed, because every effort was made to match the periods.

We conclude that the tendency to exhibit antiphase rather than synchrony at short delays is attributable to the initial region of negative slope in Type II inhibitory PRCs (Fig. 2B2) and to the scarcity of Type I PRCs combined with the vulnerability of very early synchrony in circuits with Type I PRCs to heterogeneity. There is, however, another contributing factor that relies on the discontinuity consistently observed in PRCs measured for strong inhibition [12], [23] (see Fig. 2B1 and B2). A strong inhibition applied immediately before a spike would have occurred in the absence of the inhibition (at a phase just less than one) consistently delays the next spike much more than one applied immediately after a spike (at a phase just greater than zero). Consequently, in the absence of conduction delays, synchrony is unstable; if one neuron happens to fire just before the other neuron was going to spike, the second neuron is substantially delayed and synchrony is disrupted (as illustrated in Fig. S2). Therefore simply using the slope of the PRC to determine whether synchrony will be stable [16], [23], [25] is not sufficient if the PRC is discontinuous. The addition of a short conduction delay removes this discontinuity and stabilizes synchrony. In some cases the apparent discontinuity is due to resetting that is manifested in the second [26] rather than the first cycle after the perturbation, but that is not the case here. Overall, inhibitory coupling in these neurons favors synchronous activity at shorter normalized delays (0.1 to 0.7) than excitatory coupling (>0.5). Given that all but one of the inhibitory PRCs were Type II, the most likely circuit configuration for the hybrid circuits is to be comprised of two Type II neurons, so the solution structure illustrated in Fig. 8C should predominate. In the next section, we present evidence that the solution structure illustrated in Fig. 8C does indeed predominate.

### Noise Reveals Bistability in the Transition from Very Early Antiphase to Synchrony

**Figure 9:**
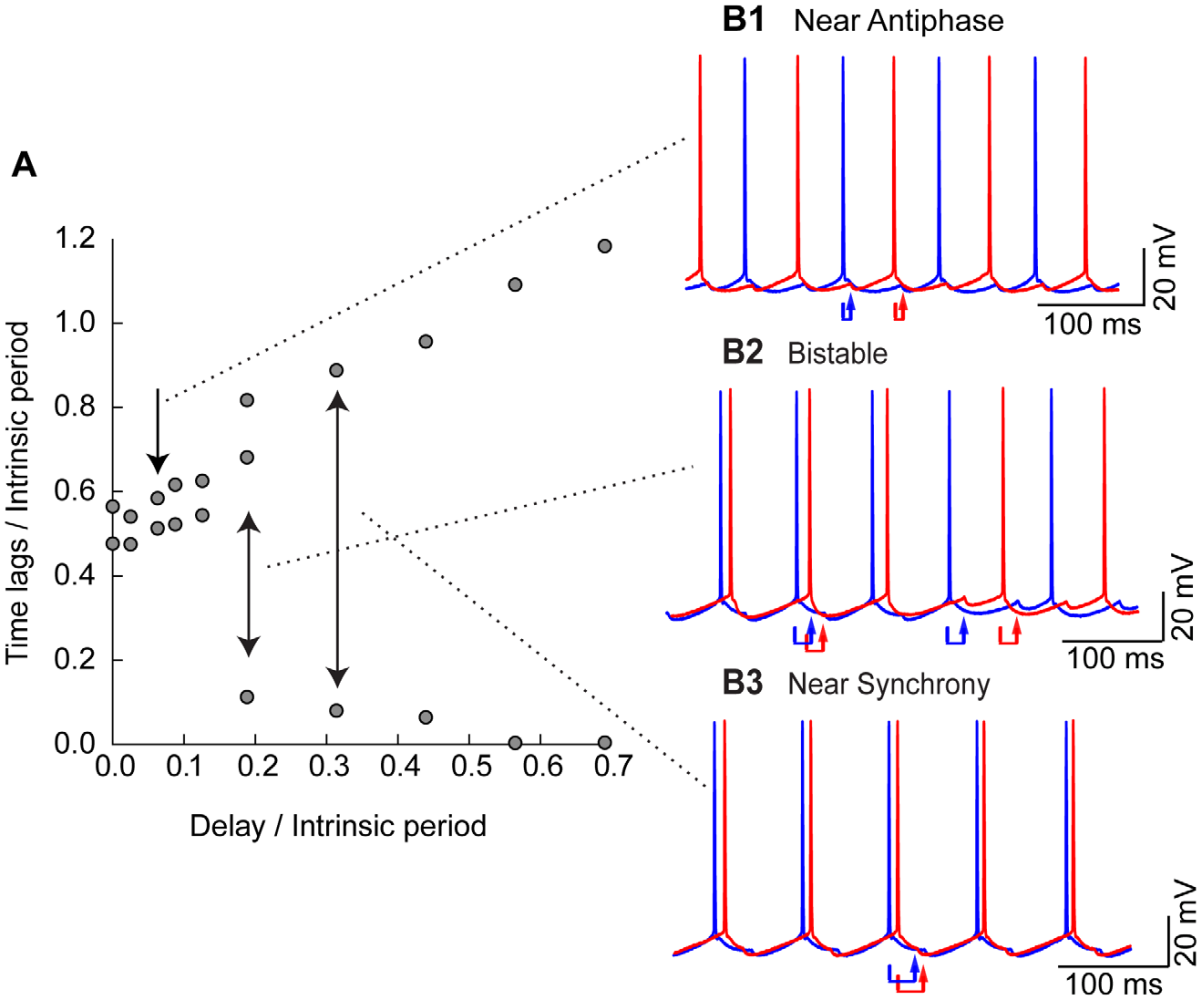
Typical firing patterns observed in inhibitory hybrid circuits. A. Representative data for a single hybrid circuit coupled with inhibition. Time lags and delays were normalized by the period of the slower neuron. B. The red and blue arrows show the delay between action potential firing in one neuron and the arrival of an input to the other neuron. B1. An antiphase mode observed at a normalized delay of 0.09 is representative of the firing patterns observed at the shortest delays. Intrinsic periods for this pair were approximately 80 ms. B2. Bistability between synchrony and anti-phase is observed at the sharp transition near a normalized delay of 0.19. B3. Near synchrony observed at a delay of 0.31.

Fig. 9A shows data from a single representative hybrid circuit. A sharp transition from near antiphase to near synchrony is observed at a normalized delay of about 0.19. This transition could occur if the circuit is comprised of two neurons with Type II PRCs as in Fig. 2B2, as the delays are increased so the locking point for synchrony acquires a positive slope. Fig. 9B1 shows the voltage traces for each of the two neurons in the hybrid circuit firing in antiphase for a short delay corresponding to antiphase at a normalized delay of 0.09. At a normalized delay of 0.19 delay (Fig. 9B2) switching between near synchrony and antiphase is observed consistent with the prediction of bistability in Fig. 8C. At a normalized delay of 0.31 near synchronous activity is observed (Fig. 9B3).

**Figure 10:**
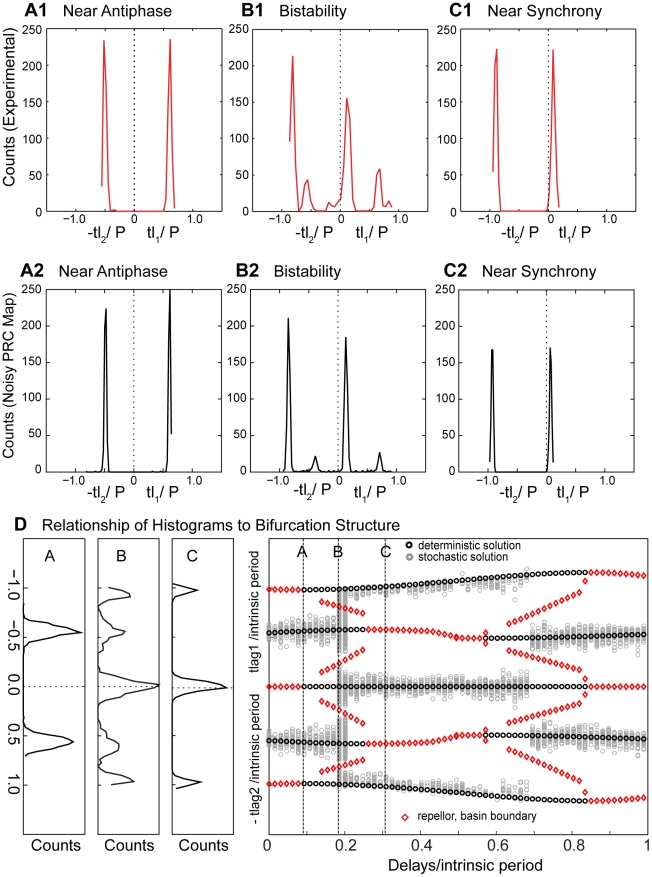
Noisy map based on the PRC exhibits bistability and shows when one bistable mode is favored over the other. Experimental histograms corresponding to the data illustrated in Fig. 9 B1, B2 and B3 with normalized delays of 0.09, 0.19 and 0.31 are shown for near antiphase (A1), bistability (B1) and near synchrony (C1) respectively. The output of the noisy map is shown for antiphase (A2), bistability (B2) and near synchrony (C2) for a hypothetical circuit constructed of two neurons with Type II inhibitory PRCs (as in Fig. 2B2), at the same delays as for the corresponding experimental data. We introduced 4% heterogeneity in the intrinsic periods in order to reproduce the asymmetry in the experimental data. D) The right side shows the predicted solution structure for the hybrid circuit composed of two identical neurons with Type II inhibitory PRCs (as in Fig. 2B2). The deterministic stable solutions are indicated by the black circles, and the unstable ones are indicated by red diamonds. The gray circles are the output of the noisy map based on the PRC. The histograms at left were constructed using the noisy map for delays corresponding to each labeled arrow on the solution structure at right, with A for near antiphase, B for bistability, and C for near synchrony. The axes of the histogram are aligned so the ordinate scale matches the time lag scale on the ordinate of the bifurcation structure. Each peak in the histograms is centered on a stable solution branch at the corresponding slice in the bifurcation diagram. For the symmetric case of two identical neurons, bistability (B) has five peaks and near synchrony (C) has three. The experimental histograms shown at the top and those produced by the noisy map and shown in the middle row have one less peak for bistability and near synchrony. This is because heterogeneity causes one neuron to lead consistently in the near synchronous mode instead of the random leader switching observed in the homogeneous circuit.

The right hand side of Fig. 10D shows the time lags (black circles) predicted for stable modes from the PRC exactly as in the homogeneous case for two cells with Type II PRCs as shown in Fig. 8C. However, here we keep track of the two time lags in the circuit separately as in the histograms in Fig. 7A and B. The black circles correspond to predicted stable modes. In this figure, we also show the predicted unstable modes (red diamonds), because they form the boundaries between bistable modes. The solution branches (black circles and red diamonds) at ±0.6 on the y-axis correspond to the antiphase mode whereas the peaks at zero and near ±1 correspond to synchrony. For normalized delays between about 0.1 and 0.25, synchrony is bistable with antiphase, but any time lags that fall between the red diamonds and the time lags for antiphase will converge to antiphase. At the beginning of the bistable regime this includes almost all time lags. As the delay is increased, the domain that converges to antiphase shrinks, and the one that converges to synchrony grows. As before, we used a noisy map based on the measured PRCs, in this case the Type II PRC shown in Fig. 2B2, and the time lags produced by the noisy map are shown as gray circles. The left part of Fig. 10D shows the histograms produced by the noisy map for two identical neurons with Type II PRCs. At a normalized delay value of 0.09 (line marked “A” in panel D) only the two peaks associated with antiphase are observed. At a normalized delay value of 0.19 (line marked “B” in panel D), there are five peaks: two that sample the antiphase mode and three that sample the synchronous mode. At a normalized delay value of 0.31 (line marked “C” in panel D), only three peaks corresponding to synchrony remain.

We compare the experimentally observed histograms (Fig. 10A1) associated with the near antiphase mode in Fig. 9B1, the bistable mode (Fig. 10B1) observed in Fig. 9B2 and the near synchronous mode (Fig. 10C1) observed in Fig. 9B3 with the corresponding histograms from Fig. 10D, with the slight difference that 4% heterogeneity in period was introduced (see Figs. 10A2,B2 and C2). The heterogeneity was introduced in order to match the asymmetry in the experimentally observed histograms. The excellent correspondence between experiments and the noisy map based on the PRC is convincing evidence that the hybrid circuit exhibits bistability and that these circuits are well characterized using only the information in the PRCs under the assumption of pulsatile coupling.

## Discussion

The main result of this study is that stable synchronization occurs when the normalized delay value falls in the positive slope region of the PRC. For the PRCs observed in this study, which are either Type I or weakly Type II, only long delays of over half the unperturbed firing period fall in this region for mutually excitatory coupling. Even for cases of coupled neurons with weakly Type II PRCs, synchronization with mutual excitation at short delays is not seen in practice, because the small, theoretical band of synchronization is obliterated by real-world factors like noise and heterogeneity in firing period. The distinction between a Type I and a weakly Type II PRC is difficult to make with confidence, and in our study the distinction between these two types is not of vital importance in the case of mutual excitation, since a little heterogeneity removes the only distinctive feature of weakly type II PRCs, which is that they promote synchrony at very short delays. With coupling delays that are over 50–70% of the unperturbed period, mutual excitation produces robust synchronization because the corresponding part of the PRC has a large positive slope and little noise. This part of the PRC is near the causal limit, for which excitatory inputs generate postsynaptic spikes with short latency. The situation is quite different for mutual inhibition. Synchrony was not observed in this study or the preceding one [12] for zero delays and mutual inhibition, but in our study synchrony was easily observed for delays ranging from 10–70% of the unperturbed period. There are three possible explanations. One is that the inhibitory PRCs are in fact weakly type II, and synchrony with zero or very short delays cannot occur because the normalized delay falls small initial region with a destabilizing negative slope. The second is that even if the PRCs are in some or all cases Type I, synchrony with short delays in circuits with Type I PRCs is quite vulnerable to heterogeneity. The third explanation (see Fig. S2) relies on the discontinuity consistently observed in PRCs measured for strong inhibition [12], [23] (see Fig. 2B1 and B2). In this latter mechanism short delays stabilize inhibitory synchrony by avoiding a destabilizing discontinuity at phase zero produced by strong coupling, and so it is distinct from that proposed by van Vreeswijk et al. [27], who showed that slowly activating inhibition could be stabilizing for reasons further explored in Achuthan et al. [28].

### Relationship to Previous Theoretical Work on Synchronization in the Presence of Delays

Much previous work on coupled oscillators with delays (see Discussion in [16]) has relied upon specific models with a specific form of coupling [19], [20], [29]–[32]. An alternative PRC-based approach based on the assumption of weak coupling [33], [34] implies that the weak coupling only slightly perturbs the intrinsic period of each oscillator, which is clearly not the case for near-causal-limit synchrony. A novel approach [9] did not presume a one to one locking between oscillators to explain gamma synchrony at a distance, but instead proposed a very specific alternate mechanism that is dependent on spike doublets that emerge as a consequence of delays and on both excitatory and inhibitory effects at both sites. Our approach reveals that the observed dynamics are very much dependent upon PRC shape and will of course vary depending upon the model and the coupling type. The neurons in the present study can be very successfully characterized as periodic oscillators if sufficient background excitation is provided. The mechanisms proposed herein for synchronization at a distance are predicated on pulsatile coupling and predictable from the PRC. Their applicability is subject to experimental verification in specific instances, but may be broadly applicable as described below.

### Relevance to Circuits within the Entorhinal Cortex

Although the superficial entorhinal cortex (EC) is a relatively well studied region of the mammalian brain, the dominant mode of communication among the EC stellate cells we study is controversial. Based on sharp-electrode recordings in brain slices, Dhillon and Jones [35] argued that EC stellate cells are not directly connected, which implies that they communicate with each other via inhibitory interneurons that introduce a polysynaptic delay. The putative effect of this delay was previously predicted to be synchronizing based upon a similar PRC-based approach [36]. More recently, Kumar and colleagues [37] used uncaging techniques to attempt to map connectivity within the superficial EC, and argued that EC stellate cells are connected directly, via excitatory synapses, with high probability. Our unpublished data collected using visual guidance and thus allowing recordings from EC stellate cells that lie very near each other, found no direct connectivity and are thus compatible with the results of Dhillon and Jones [35]. A previous study [12] showed that with no delay, mutual excitation produced synchrony whereas mutual inhibition gave rise to an antiphase mode. The present results show that these results are substantially altered by the presence of delays and support a model in which somewhat distant EC stellate cells, with polysynaptic communication delays of 5 ms or more, should synchronize best in the theta frequency band by driving inhibitory intermediaries and thus effectively inhibiting each other. Our results suggest that monosynaptic excitatory connections between stellate cells cannot support synchrony robustly, although they could support a nearly synchronous state with very small conduction delays.

In the context of the larger cortico-hippocampal circuits in which these cells participate, the longest biologically plausible delays also arise via polysynaptic pathways. For example, a direct hippocampal-prefrontal pathway has a conduction velocity of 0.6 m/s for a conduction delay of 16 ms [38], [39]. However, hippocampal activation by stimulation of the CA1 area elicited bursts in prefrontal cortex with a latency of 80 to 100 ms [40] implicating polysynaptic pathways in the delay. Resonant loops created by interconnected brain regions with accumulated transmission and activation delays on the order of 150 ms have been hypothesized to be important for the formation and retrieval of memories across cortico-hippocampal circuits [41], [42] and could contribute to phase locking at theta frequencies.

### Relevance to Other Neural Systems

Zero phase lag synchronization in the presence of presumably symmetric inter-hemispheric delays was observed between pairs of multiunit responses from area 17 in the left and right hemispheres of cats with an intact corpus callosum. The locking was disrupted when the corpus callosum was severed [43]), indicating that mutual coupling was responsible for the phase locking. The locking was at gamma frequency (40–50 Hz), and the interhemispheric delays were on the order of 4–6 ms, or about a sixth to a third of a gamma cycle. Since the projection neurons from this region are excitatory, and the type of phase resetting curves expressed by the EC cells in this study would not support locking at those delay values for excitation, we predict that the relevant PRCs for interhemispheric communication between V17 areas have a significantly different shape that the ones observed in this study. In another example, also with presumably symmetric time delays, synchronization at gamma frequency was observed with a time lag of less than a millisecond between two sites separated by up to 4 mm in hippocampal slices as a result of tetani simultaneously applied at the two sites [44]. If the conduction velocity is as slow as 300 µm/ms [45], the total delay between two hippocampal neurons 4 mm apart (including a 1 ms synaptic delay) could be 14 ms, more than half of a gamma cycle. Thus the PRCs similar to the ones observed in this study could produce such synchronization. Finally, synchronization was observed in a computational model [46] between gamma modules with similar frequencies in the presence of conduction delays up to 8 ms. For all of the cited examples, it is possible that synchronization may result from the mutual pulse coupling of oscillators; however, the oscillator may be a group of neurons rather than a single neuron. In order to apply the theoretical frame work used in this study to such cases, the relevant PRC becomes a property of the oscillatory unit rather than of an individual neuron. The conduction velocity in axons can be modulated [47], leaving open the possibility of a self-regulatory mechanism that adjusts delays to compensate for heterogeneity and to induce synchronization under the appropriate conditions.

### Generalization to Larger Networks

These results can be generalized to larger networks in several ways. First, instead of reciprocal coupling between only two oscillators, these methods may generally apply to two coupled populations if the dynamics of the population can be approximated by those of a representative neuron [25], [48], [49]. For the second type of generalization, two (or more neurons) reciprocally coupled via a central hub neuron [50], [51], like the networks of neurons presented in this study with direct reciprocal connections, possess the symmetry required for synchronization at a distance, but the robustness of this architecture to heterogeneity and noise has not yet been characterized. Finally, we can generalize to large fully connected networks with delays. Stability of the in phase synchronous state for two neurons translates to stability of the fully synchronized large network state (provided that the aggregate input received by each neuron is not too strong [25]). In the networks we studied, in the absence of delays, mutual excitation led to synchrony whereas mutual inhibition led to antiphase locking [12]. One of the most interesting aspects of our study is that the presence of delays that were a small fraction of the period inverted these results such that mutual inhibition favored synchrony whereas mutual excitation was desynchronizing.

## Methods

### Experimental Methods

#### Tissue preparation

All experimental protocols were approved by the University of Utah Institutional Animal Care and Use Committee. Horizontal sections of medial entorhinal cortex were prepared from 21 to 31 day-old Long-Evans rats of either sex. All chemicals were obtained from Sigma (St. Louis, MO) unless otherwise noted. After anesthetization with isoflurane and decapitation, brains were removed and immersed in 0°C solution consisting of the following (in mM): Sucrose (215), NaHCO_3_ (25), D-glucose (20), KCl (2.5), CaCl_2_ (0.5), NaH_2_PO_4_ (1.25), MgCl_2_ (3), buffered to pH 7.4 with 95/5% O_2_/CO_2_. Horizontal slices were cut to a thickness of 400 µm (Leica VT 1200, Leica Microsystems GMBH, Wetzlar, Germany). After the cutting procedure, slices were incubated in artificial cerebrospinal fluid (ACSF) at 30°C for 20 minutes before being cooled to room temperature (20°C). The ACSF consisted of the following (in mM): NaCl (125), NaHCO_3_ (25), D-glucose (25), KCl (2), CaCl_2_ (2), NaH_2_PO_4_ (1.25), MgCl_2_ (1), and was buffered to pH 7.4 with 95/5% O_2_/CO_2_. After the incubation period, slices were moved to the stage of an infrared differential interference contrast-equipped microscope (Axioscope 2+; Zeiss, Oberkochen, Germany). In some cases, the ACSF contained 10 µM CNQX, 50 µM picrotoxin, and 30 µM AP-5 to block ionotropic synaptic activity. For the majority of recordings, however, we did not use synaptic blockers in order to be able to measure potential synaptic connections between cells. In no case did we observe synaptic or electrical connections between cells. All recordings were conducted between 32 and 34°C.

#### Electrophysiology

Electrodes were drawn on a horizontal puller (P97; Sutter Instruments, Novato, CA) and filled with an intracellular solution consisting of the following (in mM): K-gluconate (120), KCl (20), HEPES (10), diTrisPhCr (7), Na_2_ATP (4), MgCl_2_ (2), Tris-GTP (0.3), EGTA (0.2) and buffered to pH 7.3 with KOH. Final electrode resistances were between 3 and 4 MΩ, with access resistance values between 4 and 12 MΩ. Electrophysiological recordings were performed with a current-clamp amplifier (Axoclamp 2B; Molecular Devices, Union City, CA), and data were acquired using custom software developed in Matlab (v. 2007b, Mathworks, Natick, MA) utilizing the data acquisition toolbox or custom software developed in C++ running on a Linux platform.

#### Dynamic clamp

For dynamic clamp experiments, the current-clamp amplifier was driven by an analog signal from an x86 personal computer running Real-Time Application Interface Linux and an updated version of the Real-Time Linux Dynamic Clamp [52] called Real-Time Experimental Interface [53]. For all experiments, synaptic stimuli were generated using conductances representing synaptic excitation (AMPA-like) or inhibition (GABA_A_-like): *I_AMPA_ = g_e_(t)(V_mem_−E_e_), I_GABA_ = g_i_(t)(V_mem_−E_i_)*.

The reversal potentials for excitation (E_e_) and inhibition (E_i_) were set to 0 and −75 mV, respectively. Individual synaptic events were modeled as biexponential functions. In all experiments, the rise time for individual excitatory and inhibitory events was set to 1 ms, while the decay time constant was 2 ms for excitation and 8 ms for inhibition. Individual synaptic events had a peak conductance between 0.5 and 6 nS. The sample rate of the dynamic clamp was set to10 kHz. A measured junction potential of approximately 10 mV was subtracted from all recordings and taken into account during dynamic clamp experiments. Data were collected at 10 kHz and filtered at 3 kHz.

#### Phase resetting curve measurements

Spike time response curve measurements were done in the same manner as Netoff et al. [12]. Briefly, neuronal spike rate was maintained constant for individual cells (between 8 and 12 Hz) using a spike rate controller and DC current while randomly timed individual artificial excitatory or inhibitory synaptic events were delivered every sixth cycle. The phase (φ) of a perturbation was calculated by normalizing the stimulus interval *ts* by the intrinsic period P_i_ of the component neuron involved in the experiment (Fig. 1A). P_i_ was calculated by taking the average of three interspike intervals immediately before any perturbation was given. Phase resetting was calculated by the equation f_j_(φ) = (P_j_−P_i_)/P_i_, where P_j_ is the length of the cycle that contains the perturbation. All phase resetting measurements were then evenly divided into 100 bins such that each bin had at least 3 phase resetting values, and the mean and variance were calculated for each bin. If any single bin contained less than three phase resetting values, that bin was expanded to include immediately neighboring bins on both the left and right sides and an average of the phase resetting values in all the three bins was taken instead. A phase resetting curve (PRC) was then obtained by a 2nd, 3rd or 4th^th^ order polynomial fit to the mean phase resetting of each bin. If the least square fit to a second order polynomial (a single peak) had less least squares error than a higher order fit, the PRC was classified as Type I, otherwise it was classified as Type II. For the Type I excitatory PRCs, there was a very small region of very small delays at very late phases. Advances longer than the time expected to the next spike cannot be observed in practice, which biases the data at very late phases toward delays [54], [55], therefore these small delays were considered spurious and ignored.

#### Two cell recordings

For two cell recordings pairs of cells were patched and recordings were taken simultaneously. In most cases neurons were within 100 µm of each other. Firing rate was set to a value between 8 and 12 Hz before coupling using DC current. After setting firing rate, neurons were connected reciprocally through artificial synaptic connections using dynamic clamp. Synaptic input in the post-synaptic neuron was triggered via a spike detector in the pre-synaptic cell. Artificial synaptic waveforms used during artificial coupling experiments were the same as those used to measure phase resetting curves. Spike detection was based on a simple threshold crossing of membrane voltage (−20 mV). For conditions implementing a delay, synaptic activity in the post-synaptic cell was delayed relative to spike detection by a user defined value.

### Theoretical Methods

#### Noisy iterated pulse coupled map

In contrast to the pulse coupled maps used by Netoff et al. [12] and Sieling et al. [24], the map used in this study includes conduction delays (first included in such a map in [56], such that the pulse emission (spiking) and receipt of a pulse (the EPSP) occur at different times so that there are two classes of events. The map has no predetermined firing order, but instead the phases of each neuron are updated as each event occurs. The intrinsic period P_j_ of each neuron, the PRC for each neuron, the conduction delay times τ _i,j_ from neuron i to neuron j, and the initial phase φ_j_[0] are sufficient to determine, under the assumption of pulsatile coupling, all the firing times in the future. The next event is determined by finding the smallest interval remaining until either the next spike or the next arrival of an input at its destination. The interval until the next spike in neuron j barring the receipt of any other inputs is P_j_−P_j_φ_j_ whereas the interval until the each input reaches its destination is e_q,i_+τ_i,j_−t where t is the current elapsed time and e_q,i_ refers to the time of pulse emission for pulses indexed by q from each neuron i that have not yet reached the other neuron j. When a neuron fires, its phase is reset to zero, and the emission time is stored in a queue until it is cleared by arrival at its destination after a conduction delay. When an input is received, the phase resetting due to that input is subtracted from the current phase. This map is a reduction of the full dynamics of a system to an ideal pulse coupled system. The map representing an ideal pulse coupled system with delays was implemented in C code.

In order to simulate the biological noise level, noise was added to the phase resetting received by each neuron when an input was received. The noise was taken from a Gaussian distribution with a mean and variance equal to that of the measured PRC. The PRC (n = 100) was binned (bin size  = 100) and the standard deviation, σ, was measured at the center of every bin. The upper and lower envelopes of the PRC were constructed by applying polynomial fits to the mean PRC ±σ. We typically used the same order of the polynomials used to fit the mean PRC for the envelopes as in Fig. 1C. The algorithm was constrained to reject any phase resetting values that violated causality by causing a spike to occur before the input that reset it.

#### Circular statistics

Circular statistics [57] were used in order to quantify the effect of noise on the strength of phase locking. Using this method the mean and variability of the network phase can be represented as a single vector. The angle of the vector represents the mean network phase (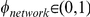) and is denoted by,
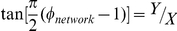
where X and Y are,
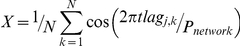
and
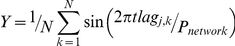
where N is the number of cycles recorded in the experiments for neuron *j*, tlag _j,k_ is the time lag for the k^th^ cycle of neuron j and denotes the interspike interval between the two neurons and P_network_ is the average period of the network. The length of the vector R represents the strength of the phase locking where R^2^ = X^2^+Y^2^.

## Supporting Information

Figure S1 **Explanation of stable versus unstable intersections in the graphical method.** A point on the curves in panels A and B can be plotted at each phase for each neuron because the stimulus interval for each phase in one neuron determines the next recovery interval in the same neuron. Panel C shows that for a fixed delay value, the recovery interval for one neuron determines the next stimulus interval in the partner neuron. Therefore we can construct a map of the intervals that result as the network is perturbed away from an intersection point. Because of how the axes are set up in panels A and B, the next interval can always be determined by moving vertically from the black curve to the red curve, or horizontally from the red curve to the black curve. Panel A shows that this implies that if the black curve is steeper at the intersection, trajectories return to the intersection when displaced, whereas part B shows that if the red curve is steeper at the intersection, they do not. This proof is for a k value equal to one, but the principle applies to higher values.
(TIF)

Figure S2 **Explanation of how a discontinuity in the PRC between 0 and 1 destabilizes synchrony.** The first two spikes are synchronous, and the third synchronous pair of spikes should have occurred at the time indicated by the red dashed line. For a discontinuous PRC in which f(1)>f(0), any perturbation Δt from synchrony causes one neuron to fire too early, after an interval equal to Pi+Pif(0)−Δt. The partner neuron receives two inputs (one at zero phase and one at an interval of Δt before it was to reach a phase of one and fire) that delay the next spike in the second neuron until an interval after the synchronous spike of Pi+Pif(0)+Pif(1)−f′(1) Δt. This delay causes the first neuron to receive an input later in the cycle with a stimulus interval that can be obtained by subtraction of the short interval in the first neuron from the long interval in the second neuron. Clearly the discontinuity causes perturbations from synchrony to grow, rendering synchrony unstable.
(TIF)

Text S1 **Derivation of nonzero time lag in synchrony perturbed by heterogeneity.** This file contains the details of the derivation of the equation in the section “Effects of heterogeneity on synchrony: theoretical results” with the terms as illustrated in Fig. 6B.
(DOC)
