# Supplementary figures and images for: Short Conduction Delays Cause Inhibition Rather than Excitation to Favor Synchrony in Hybrid Neuronal Networks of the Entorhinal Cortex
Source: PLoS Comput Biol. 2012 Jan 5;8(1):e1002306. doi: 10.1371/journal.pcbi.1002306 (PMC3252263; doi:10.1371/journal.pcbi.1002306)

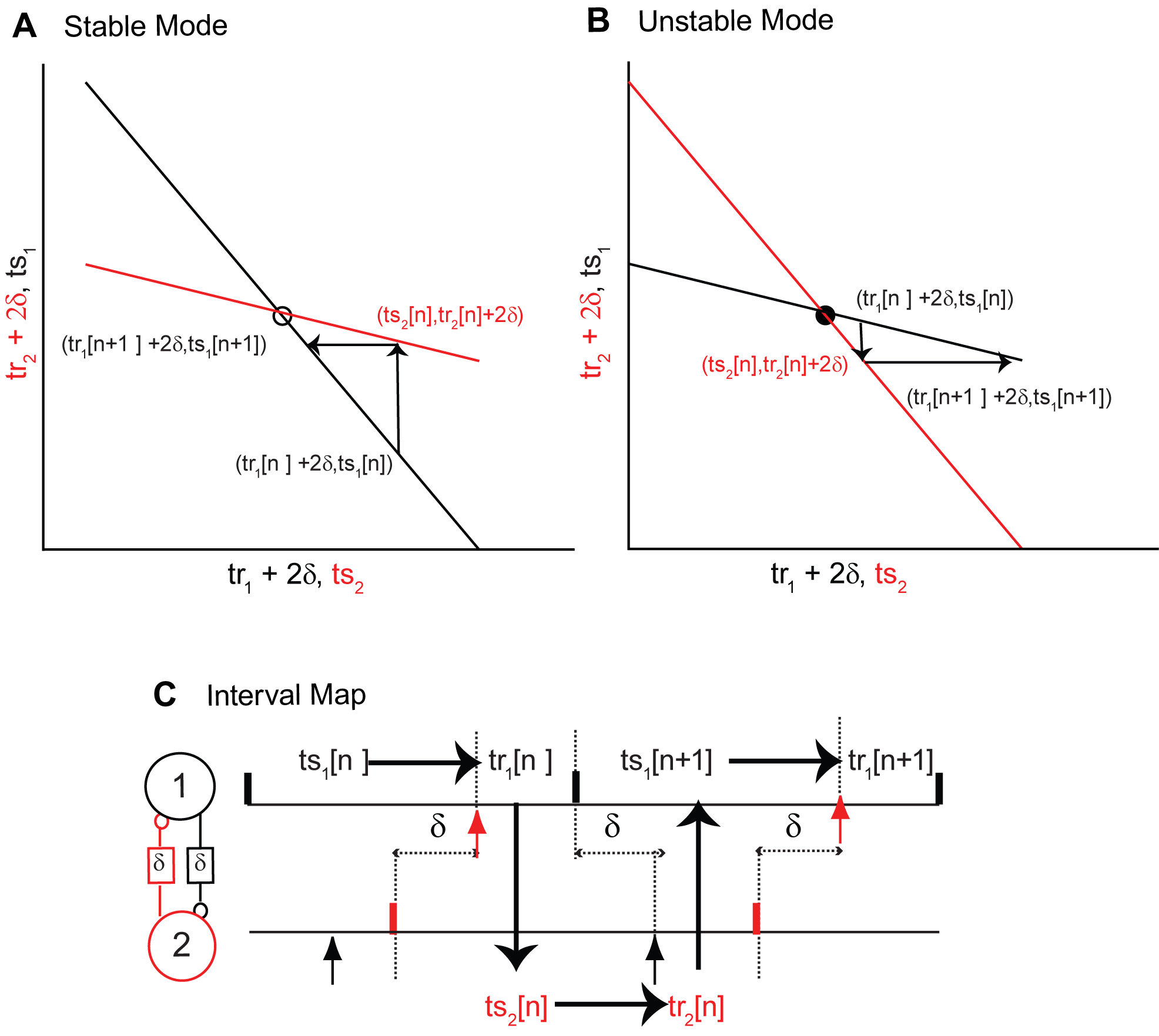

Supplement: Figure S1 — Explanation of stable versus unstable intersections in the graphical method. A point on the curves in panels A and B can be plotted at each phase for each neuron because the stimulus interval for each phase in one neuron determines the next recovery interval in the same neuron. Panel C shows that for a fixed delay value, the recovery interval for one neuron determines the next stimulus interval in the partner neuron. Therefore we can construct a map of the intervals that result as the network is perturbed away from an intersection point. Because of how the axes are set up in panels A and B, the next interval can always be determined by moving vertically from the black curve to the red curve, or horizontally from the red curve to the black curve. Panel A shows that this implies that if the black curve is steeper at the intersection, trajectories return to the intersection when displaced, whereas part B shows that if the red curve is steeper at the intersection, they do not. This proof is for a k value equal to one, but the principle applies to higher values. (TIF) [file pcbi.1002306.s001.tif]

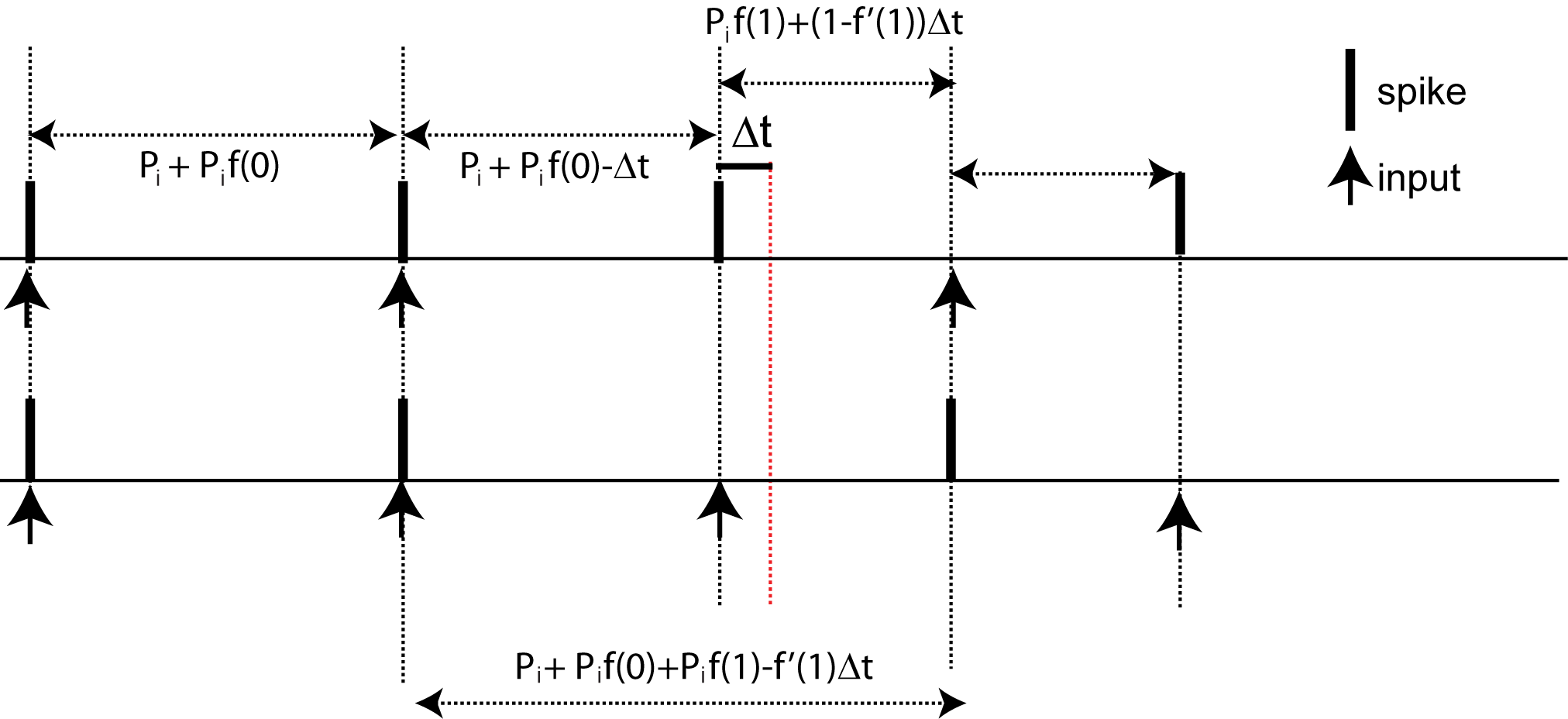

Supplement: Figure S2 — Explanation of how a discontinuity in the PRC between 0 and 1 destabilizes synchrony. The first two spikes are synchronous, and the third synchronous pair of spikes should have occurred at the time indicated by the red dashed line. For a discontinuous PRC in which f(1)>f(0), any perturbation Δt from synchrony causes one neuron to fire too early, after an interval equal to Pi+Pif(0)−Δt. The partner neuron receives two inputs (one at zero phase and one at an interval of Δt before it was to reach a phase of one and fire) that delay the next spike in the second neuron until an interval after the synchronous spike of Pi+Pif(0)+Pif(1)−f′(1) Δt. This delay causes the first neuron to receive an input later in the cycle with a stimulus interval that can be obtained by subtraction of the short interval in the first neuron from the long interval in the second neuron. Clearly the discontinuity causes perturbations from synchrony to grow, rendering synchrony unstable. (TIF) [file pcbi.1002306.s002.tif]
