# Supplementary material for: Short Conduction Delays Cause Inhibition Rather than Excitation to Favor Synchrony in Hybrid Neuronal Networks of the Entorhinal Cortex
Source: PLoS Comput Biol. 2012 Jan 5;8(1):e1002306. doi: 10.1371/journal.pcbi.1002306 (PMC3252263; doi:10.1371/journal.pcbi.1002306)
Supplement: Text S1 — Derivation of nonzero time lag in synchrony perturbed by heterogeneity. This file contains the details of the derivation of the equation in the section “Effects of heterogeneity on synchrony: theoretical results” with the terms as illustrated in Fig. 6B. (DOC) [file pcbi.1002306.s003.doc]

**Derivation of nonzero time lag in synchrony perturbed by heterogeneity**

Assume that two neurons have the same PRC but slightly different values of intrinsic period P_1_ and P_2_ and/or delay *δ_1_* and *δ_2_*. We then define δ as the average of *δ_1_* and *δ_2_* and Δδ = *δ_1_* –*δ_2._* If each neuron were coupled to an identical partner, then by symmetry a synchronous mode exists with ts_i_ = δ_i_/P_i_. We also assume the PRC is linear in the vicinity of δ/P_1_ and δ/P_2_. We then define ε as the positive, non-zero time lag between the firing of the two coupled heterogeneous neurons in a nearly synchronous mode resulting from the perturbation of synchrony in the homogeneous networks.

Therefore the stimulus intervals can now be expressed in terms of delays and the near zero time lag ε (see Fig. 6B):

$$ts_{1}[\infty]=\delta_{2}+\varepsilon=\delta+\Delta\delta/2+\varepsilon$$

$$ts_{2}[\infty]=\delta_{1}-\varepsilon=\delta-\Delta\delta/2-\varepsilon$$

The stimulus and recovery intervals from either neuron add up to the network period:

$$ts_{1}[\infty]+tr_{1}[\infty]=ts_{2}[\infty]+tr_{2}[\infty]$$

Substituting the expressions for the stimulus intervals ts_1_[∞] and ts_2_[∞] we obtain

$$\delta+\varepsilon+\Delta\delta/2+tr_{1}[\infty]=\delta-\varepsilon-\Delta\delta/2+tr_{2}[\infty]$$

which implies that the difference between the two recovery intervals is

$$tr_{2}[\infty]-tr_{1}[\infty]=2\varepsilon+\Delta\delta$$

for identical delays. Using

$$ts_{i}[\infty]+tr_{i}[\infty]=P_{i}+P_{i}f(ts_{i}[\infty]/P_{i})$$

where *f* indicates the PRC function and substituting for the recovery intervals in terms of the stimulus intervals produces:

$$P_{2}-ts_{2}[\infty]+P_{2}f(ts_{2}[\infty]/P_{2}))-P_{1}+ts_{1}[\infty]-P_{1}f(ts_{1}[\infty]/P_{1}))=2\varepsilon+\Delta\delta$$

We can re-arrange terms and substitute for the stimulus intervals in the expressions for phase resetting to obtain:

$$P_{2}-P_{1}+(ts_{1}[\infty]-ts_{2}[\infty])+P_{2}f(\frac{\delta-\varepsilon-\Delta\delta/2}{P_{2}})-P_{1}f(\frac{\delta+\varepsilon+\Delta\delta/2}{P_{1}})=2\varepsilon+\Delta\delta$$

Since the difference between the two stimulus intervals $ts_{1}$[∞] - $ts_{2}$[∞]=$2\varepsilon+\Delta\delta$, we substitute it in the above equation and then linearize the PRC about *δ/P_i_* to obtain:

$$P_{2}-P_{1}+P_{2}f(\delta/P_{2})-(\varepsilon+\Delta\delta/2)f'(\delta/P_{2})-P_{1}f(\delta/P_{1})-(\varepsilon+\Delta\delta/2)f'(\delta/P_{1})=0$$

Solving for $\varepsilon$ from above equation gives us the following expression for the near zero time lag:
